# Supplementary material for: Associations between gestational weight gain under different guidelines and adverse birth outcomes: A secondary analysis of a randomized controlled trial in rural western China
Source: PLOS Glob Public Health. 2024 Jan 8;4(1):e0002691. doi: 10.1371/journal.pgph.0002691 (PMC10773947; doi:10.1371/journal.pgph.0002691)
Supplement: S1 Table — (DOCX) [file pgph.0002691.s001.docx]

S1 Table. Different recommendation ranges for gestational weight gains.

| recommendations | Total GWG (kg) | | | | Incremental weight gain during the second and third trimesters (kg/week) | | | |
| --- | --- | --- | --- | --- | --- | --- | --- | --- |
|  | underweight | Normal weight | overweight | obesity | underweight | Normal weight | overweight | obesity |
| IOM^a^[1] | 12.5-18 | 11.5-16 | 7-11.5 | 5-9 | 0.51 (0.44-0.58) | 0.42 (0.35-0.50) | 0.28 (0.23-0.33) | 0.22 (0.17-0.27) |
| Zhang et al^b^[2] | 12.8-17.10 | 12.1-16.4 | 10.4-14.9 |  |  |  |  |  |
| Sun et al^b^[3] | 8.0-11.9 | 12-13.9 | 8-9.9 | <8 |  |  |  |  |
| CNS^b^[4] | 11.0-16.0 | 8.0-14.0 | 7.0-11.0 | 5.0-9.0 | 0.46 (0.37-0.56) | 0.37 (0.26-0.48) | 0.30 (0.22-0.37) | 0.22 (0.15-0.30) |
| NHC^b^[5] | 11.0-16.0 | 8.0-14.0 | 7.0-11.0 | 5.0-9.0 | 0.46 (0.37-0.56) | 0.37 (0.26-0.48) | 0.30 (0.22-0.37) | 0.22 (0.15-0.30) |

^a^preconception BMI categories defined as: underweight (<18.5 kg/m^2^), normal weight (18.5-24.9 kg/m^2^), overweight (25.0-29.9 kg/m^2^), obesity (≥30 kg/m^2^).

^b^preconception BMI categories defined as: underweight (<18.5 kg/m^2^), normal weight (18.5-23.9 kg/m^2^), overweight (24.0-27.9 kg/m^2^), obesity (≥28 kg/m^2^).

Reference:

1. Rasmussen KM, Yaktine AL, Institute of Medicine (U.S.), editors. Weight gain during pregnancy: reexamining the guidelines. Washington, DC: National Academies Press; 2009.

2. Zhang CX, Lai JQ, Liu KY, Yang NH, Zeng G, Mao LM, et al. Optimal gestational weight gain in Chinese pregnant women by Chinese-specific BMI categories: a multicentre prospective cohort study. Public Health Nutr. 2021;24:3210-3220.

3. Sun Y, Shen Z, Zhan Y, Wang Y, Ma S, Zhang S, et al. Investigation of optimal gestational weight gain based on the occurrence of adverse pregnancy outcomes for Chinese women: a prospective cohort study. Reprod Biol Endocrinol RBE. 2021;19:130.

4. Chinese Nutrition Society. Weight monitoring and evaluation during pregnancy period of Chinese women: group standard T/CNSS 009-2021. Accessed Septermber 3, 2021. https://www.cnsoc.org/ otherNotice/392100200.html

5. National Health Commission of the People's Republic of China. Standard of Recommendation for Weight Gain during Pregnancy Period: industry standard WS/T 801-2022. Accessed August 18,2022. http://www.nhc.gov.cn/wjw/fyjk/202208/864ddc16511148819168305d3e576de9.shtml
