# Supplementary material for: Associations between gestational weight gain under different guidelines and adverse birth outcomes: A secondary analysis of a randomized controlled trial in rural western China
Source: PLOS Glob Public Health. 2024 Jan 8;4(1):e0002691. doi: 10.1371/journal.pgph.0002691 (PMC10773947; doi:10.1371/journal.pgph.0002691)
Supplement: S2 Table — (DOCX) [file pgph.0002691.s002.docx]

S2 Table. Association between different GWG classifications and birth weight and gestational age among pregnant women with normal weight based on Chinese guidelines (n=1,239)

|  | IOM category^a^ | | NHC category^a^ | | z-score category 1^ab^ | | z-score category 2^ac^ | |
| --- | --- | --- | --- | --- | --- | --- | --- | --- |
|  | Inadequate | Excessive | Inadequate | Excessive | Below average | Above average | Below average | Above average |
| Birth weight | -48.84  (-109.61, 11.93) | -3.80  (-79.78, 72.18) | -29.69  (-81.26, 21.88)) | 38.62  (-26.25, 103.50) | -41.42  (-92.97, 10.12) | 21.71  (-75.46, 118.88) | -40.76  (-88.70, 7.18) | 8.51  (-102,71, 119.73) |
| Birthweight for gestational age z-score | -0.11  (-0.26, 0.03) | -0.04  (-0.23, 0.14) | -0.06  (-0.19, 0.07) | 0.05  (-0.11, 0.21) | -0.07  (-0.19, 0.06) | 0.05  (-0.19, 0.29) | -0.09  (-0.20, 0.03) | 0.02  (-0.25, 0.29) |
| Gestational age at birth | 0.01  (-0.22, 0.25) | 0.07  (-0.23, 0.36) | 0.01  (-0.19, 0.21) | 0.10  (-0.15, 0.34) | -0.06  (-0.26, 0.14) | 0.06  (-0.31, 0.43) | -0.01  (-0.19, 0.18) | 0.05  (-0.37, 0.48) |

Abbreviations: GWG, gestational weight gain; IOM, Institute of Medicine; NHC, National Health Commission.

^a^Data are presented with adjusted odd ratios and 95% confidence intervals by performing generalized linear models. The adjustments included parental education, occupation and age, maternal parity, the gestational week during early trimester when the maternal weight was measured, mid-upper arm circumference, randomized regimens and pre-pregnant disease history, household wealth at enrollment and infant sex.

^b^Category 1 refers to subjects classified into three groups by percentiles of z-score(z-score percentile<25^th^, 25^th^ to 75^th^, >75^th^). We set subgroup 2 (25th to 75th) as the reference group.

^c^Category 2 refers to subjects classified into three groups by of z-score (< -1, -1 to 1, >1). We set subgroup 2 (-1 to 1 SD) as the reference group.
