# Supplementary material for: Associations between gestational weight gain under different guidelines and adverse birth outcomes: A secondary analysis of a randomized controlled trial in rural western China
Source: PLOS Glob Public Health. 2024 Jan 8;4(1):e0002691. doi: 10.1371/journal.pgph.0002691 (PMC10773947; doi:10.1371/journal.pgph.0002691)
Supplement: S3 Table — (DOCX) [file pgph.0002691.s003.docx]

S3 Table. AUC for gestational weight gain recommendation range corresponding to each adverse birth outcome, under IOM, NHC, and z-score, respectively.

|  | AUC (95% CI) | | | | P value |
| --- | --- | --- | --- | --- | --- |
|  | IOM category | NHC category | z-score category 1^a^ | z-score category 2^b^ |  |
| Preterm birth | 0.6683 (0.5954, 0.7412) | 0.6706 (0.5974, 0.7438) | 0.6706 (0.5975, 0.7436) | 0.6737 (0.6008, 0.7465) | 0.80 |
| Post-term birth | 0.6162 (0.5603, 0.6720) | 0.6334 (0.5810, 0.6857) | 0.6167 (0.5623, 0.6711) | 0.6249 (0.5695, 0.6802) | 0.55 |
| LBW | 0.7242 (0.6467, 0.8016) | 0.7250 (0.6482, 0.8017) | 0.7225 (0.6438, 0.8011) | 0.7220 (0.6441, 0.7999) | 0.92 |
| Macrosomia | 0.7794 (0.6891, 0.8698) | 0.8058 (0.7202, 0.8914) | 0.7868 (0.6856, 0.8780) | 0.7718 (0.6746, 0.8690) | 0.54 |
| SGA | 0.6438 (0.6009, 0.6866) | 0.6455 (0.6027, 0.6883) | 0.6404 (0.5794, 0.6834) | 0.6412 (0.5983, 0.6842) | 0.56 |
| LGA | 0.6953 (0.6298, 0.7608) | 0.7049 (0.5414, 0.7684) | 0.7038 (0.6371, 0.7706) | 0.6962 (0.6289, 0.7636) | 0.73 |

Abbreviations: AUC, areas under the curve; IOM, Institute of Medicine; NHC, National Health Commission; LBW, low birth weight; SGA, small-for-gestational-age; LGA, large-for-gestational-age.

^a^Z-score category 1 refers to subjects classified into three groups by percentiles of z score (z-score percentile<25^th^, 25^th^ to 75^th^, >75^th^).

^b^Z-score category 2 refers to subjects classified into three groups by of z score (< -1, -1 to 1, >1).
