# Supplementary material for: Associations between gestational weight gain under different guidelines and adverse birth outcomes: A secondary analysis of a randomized controlled trial in rural western China
Source: PLOS Glob Public Health. 2024 Jan 8;4(1):e0002691. doi: 10.1371/journal.pgph.0002691 (PMC10773947; doi:10.1371/journal.pgph.0002691)
Supplement: S4 Table — (DOCX) [file pgph.0002691.s004.docx]

S4 Table. Association between different GWG and birth outcomes among pregnant women with normal weight based on Chinese guidelines (n=1,239).

|  | Weekly gains during the second and third trimesters^a^ | | Weight-gain-for-gestational age z-score^a^ | |
| --- | --- | --- | --- | --- |
|  | Mean difference (95% CI) / OR (95% CI) | *P* | Mean difference (95% CI) / OR (95% CI) | *P* |
| Birth weight | 79.81 (-18.19, 177.82) | 0.11 | 21.57 (3.48, 39.67) | 0.02 |
| Birthweight for gestational age z-score | 0.18 (-0.06, 0.42) | 0.15 | 0.05 (0.01, 0.10) | 0.02 |
| Gestational age at birth | -0.05 (-0.43, 0.32) | 0.79 | -0.01 (-0.08, 0.06) | 0.79 |
|  |  |  |  |  |
| Preterm birth | 1.63 (0.48, 5.53) | 0.44 | 1.02 (0.80, 1.30) | 0.85 |
| Post-term birth | 0.37 (0.12, 1.16) | 0.09 | 0.89 (0.74, 1.07) | 0.22 |
| LBW | 0.72 (0.19, 2.70) | 0.63 | 0.94 (0.73, 1.19) | 0.60 |
| Macrosomia | 2.49 (0.48, 12.87) | 0.28 | 1.30 (0.89, 1.90) | 0.18 |
| SGA | 0.86 (0.41, 1.79) | 0.69 | 0.96 (0.84, 1.10) | 0.56 |
| LGA | 1.87 (0.64, 5.41) | 0.25 | 1.28 (1.01, 1.61) | 0.04 |

Abbreviations: GWG, gestational weight gain; OR, odds ratio; LBW, low birth weight; SGA, small-for-gestational-age; LGA, large-for-gestational-age.

^a^Data are presented with adjusted mean difference or adjusted odd ratios and their 95% confidence intervals by performing generalized linear models. The adjustments included parental education, occupation and age, maternal parity, the gestational week during early trimester when the maternal weight was measured, mid-upper arm circumference, randomized regimens and pre-pregnant disease history, household wealth at enrollment and infant sex.
