# Supplementary material for: Associations between gestational weight gain under different guidelines and adverse birth outcomes: A secondary analysis of a randomized controlled trial in rural western China
Source: PLOS Glob Public Health. 2024 Jan 8;4(1):e0002691. doi: 10.1371/journal.pgph.0002691 (PMC10773947; doi:10.1371/journal.pgph.0002691)
Supplement: S5 Table — (DOCX) [file pgph.0002691.s005.docx]

S5 Table. Classification of weekly average gestational weight gain during the second and third trimesters and consistency among different guidelines based on complete eligible data (n=1,566).

| Measure | N | GWG categories [n(%)] | | | Kappa |
| --- | --- | --- | --- | --- | --- |
|  |  | Inadequate^a^ | Adequate^a^ | Excessive^a^ |  |
| IOM 2009 | 1566 | 1009(64.4) | 278(17.8) | 279(17.8) | Ref. |
| NHC 2022 | 1566 | 756(48.3) | 503(32.1) | 307(19.6) | 0.70 |

Abbreviations: GWG, gestational weight gain; IOM, Institute of Medicine; NHC, National Health Commission.

^a^Inadequate, adequate, and excessive GWG were defined as below, within and above the recommended range.
