# Supplementary material for: Associations between gestational weight gain under different guidelines and adverse birth outcomes: A secondary analysis of a randomized controlled trial in rural western China
Source: PLOS Glob Public Health. 2024 Jan 8;4(1):e0002691. doi: 10.1371/journal.pgph.0002691 (PMC10773947; doi:10.1371/journal.pgph.0002691)
Supplement: S7 Table — (DOCX) [file pgph.0002691.s007.docx]

S7 Table. Interaction P values between GWG categories and parental education and infant sex for adverse birth outcomes.

|  | IOM category | NHC category | z-score category 1 | z-score category 2 |
| --- | --- | --- | --- | --- |
| Preterm birth |  |  |  |  |
| P_interaction_^a^ | 0.03 | 0.06 | 0.02 | 0.07 |
| P_interaction_^b^ | 0.57 | 0.32 | 0.72 | 0.91 |
| P_interaction_^c^ | 0.94 | 0.89 | 0.43 | 0.97 |
| Post-term birth |  |  |  |  |
| P_interaction_^a^ | 0.73 | 0.30 | 0.84 | 0.54 |
| P_interaction_^b^ | 0.89 | 0.96 | 0.32 | 0.64 |
| P_interaction_^c^ | 0.62 | 0.45 | 0.52 | 0.13 |
| LBW |  |  |  |  |
| P_interaction_^a^ | 0.36 | 0.99 | 0.47 | 0.82 |
| P_interaction_^b^ | 0.33 | 0.22 | 0.47 | 0.33 |
| P_interaction_^c^ | 0.74 | 0.94 | 0.53 | 0.94 |
| Macrosomia |  |  |  |  |
| P_interaction_^a^ | 0.53 | 0.73 | 0.93 | 0.68 |
| P_interaction_^b^ | 0.06 | 0.19 | 0.09 | 0.19 |
| P_interaction_^c^ | 0.21 | 0.29 | 0.45 | 0.28 |
| SGA |  |  |  |  |
| P_interaction_^a^ | 0.77 | 0.87 | 0.98 | 0.88 |
| P_interaction_^b^ | 0.55 | 0.94 | 0.36 | 0.59 |
| P_interaction_^c^ | 0.97 | 0.61 | 0.65 | 0.28 |
| LGA |  |  |  |  |
| P_interaction_^a^ | 0.08 | 0.12 | 0.86 | 0.19 |
| P_interaction_^b^ | 0.78 | 0.70 | 0.20 | 0.21 |
| P_interaction_^c^ | 0.95 | 0.94 | 0.95 | 0.95 |

Abbreviations: GWG, gestational weight gain; IOM, Institute of Medicine; NHC, National Health Commission; LBW, low birth weight; SGA, small-for-gestational-age; LGA, large-for-gestational-age.

^a^*P* values of interaction between GWG categories and maternal education.

^b^*P* values of interaction between GWG categories and paternal education.

^c^*P* values of interaction between GWG categories and adolescent sex.
