# Supplementary material for: Associations between gestational weight gain under different guidelines and adverse birth outcomes: A secondary analysis of a randomized controlled trial in rural western China
Source: PLOS Glob Public Health. 2024 Jan 8;4(1):e0002691. doi: 10.1371/journal.pgph.0002691 (PMC10773947; doi:10.1371/journal.pgph.0002691)
Supplement: S8 Table — (DOCX) [file pgph.0002691.s008.docx]

S8 Table. Comparison of baseline characteristics between mother-infant pairs included into final analysis and those were not.

| Factors | Included in final analysis | Not included in the final analysis | *P* |
| --- | --- | --- | --- |
| N | 1239 | 3365 |  |
| Maternal age (years) /Mean (SD) | 24.5 (4.5) | 24.7 (4.3) | 0.12 |
| Maternal education /N (%) |  |  | 0.04 |
| < 3 years | 63 (5.1) | 201 (6.0) |  |
| Primary | 301 (24.4) | 908 (27.1) |  |
| Secondary | 670 (54.2) | 1786 (53.3) |  |
| High school and above | 202 (16.3) | 457 (13.6) |  |
| Maternal occupation /N (%) |  |  | <0.001 |
| Farmer | 1000 (81.2) | 2568 (76.5) |  |
| Others | 231 (18.8) | 787 (23.5) |  |
| Parity at enrollment /N (%) |  |  | 0.63 |
| 0 | 817 (65.9) | 2183 (64.8) |  |
| 1 | 378 (30.5) | 1049 (31.2) |  |
| ≥2 | 44 (3.5) | 135 (4.0) |  |
| Maternal MUAC (cm) /N (%) |  |  | <0.001 |
| <21.5 | 171 (13.9) | 664 (20.0) |  |
| ≥21.5 | 1062 (86.1) | 2656 (80.0) |  |
| Randomized regimens /N (%) |  |  | 0.06 |
| Folic acid | 456 (36.8) | 1182 (35.1) |  |
| Folic acid plus iron | 421 (34.0) | 1078 (32.0) |  |
| Multiple micronutrient | 362 (29.2) | 1107 (32.9) |  |
| Pre-pregnancy medical history /N (%) |  |  | 0.95 |
| Yes | 242 (19.7) | 651 (19.6) |  |
| No | 985 (80.3) | 2665 (80.4) |  |
| Household wealth at enrollment^a^ /N (%) |  |  |  |
| Low | 376 (30.4) | 1106 (32.8) | 0.03 |
| Medium | 418 (33.7) | 1191 (35.4) |  |
| High | 445 (35.9) | 1070 (31.8) |  |
| Offspring sex /N (%) |  |  | 0.73 |
| Male | 693 (55.9) | 1864 (55.4) |  |
| Female | 546 (44.1) | 1503 (44.6) |  |
| Infant birth weight (gram) /Mean (SD) | 3189 (411) | 3184 (434) | 0.73 |
| Infant gestational age at birth/Mean (SD) | 39.8 (1.5) | 39.8 (1.8) | 0.71 |

Abbreviations: SD: standard deviations; MUAC, mid-upper arm circumference.

^a^Household wealth at enrollment was derived from principal component analysis for household assets and dwelling characteristics, and was further categorized by its terciles.
