# Supplementary material for: Associations between gestational weight gain under different guidelines and adverse birth outcomes: A secondary analysis of a randomized controlled trial in rural western China
Source: PLOS Glob Public Health. 2024 Jan 8;4(1):e0002691. doi: 10.1371/journal.pgph.0002691 (PMC10773947; doi:10.1371/journal.pgph.0002691)
Supplement: S9 Table — (DOCX) [file pgph.0002691.s009.docx]

S9 Table. Sensitivity analysis using inverse probability weighting to assess the potential of selection bias of the association between different GWG classifications and adverse birth outcomes among women with underweight, normal, overweight or obesity (n=1566).

|  | IOM category^a^ | | NHC category^a^ | |
| --- | --- | --- | --- | --- |
|  | Inadequate | Excessive | Inadequate | Excessive |
| Preterm birth | 1.16 (0.55, 2.43) | 1.16 (0.452.98) | 1.36 (0.69, 2.70) | 1.20 (0.51, 2.83) |
| Post-term birth | 2.99 (1.30, 6.87) | 1.80 (0.66, 4.92) | 2.37 (1.31, 4.30) | 1.30 (0.60, 2.85) |
| LBW | 1.20 (0.51, 2.81) | 0.94 (0.33, 2.70) | 1.05 (0.54, 2.04) | 0.74 (0.31, 1.80) |
| Macrosomia | 2.65 (0.56, 12.53) | 3.13 (0.57, 17.13) | 1.09 (0.30, 3.97) | 1.86 (0.50, 6.93) |
| SGA | 1.27 (0.82, 1.97) | 1.16 (0.68, 2.01) | 1.20 (0.83, 1.73) | 0.99 (0.62, 1.59) |
| LGA | 1.18 (0.5, 2.55) | 2.22 (0.97, 5.08) | 1.05 (0.54, 2.03) | 2.18 (1.12, 4.25) |

Abbreviations: GWG, gestational weight gain; IOM, Institute of Medicine; NHC, National Health Commission; LBW, low birth weight; SGA, small-for-gestational-age; LGA, large-for-gestational-age; NA, not applicable.

^a^Data are presented with adjusted odd ratios and 95% confidence intervals by performing generalized linear models. The adjustments included parental education, occupation and age, maternal parity, the gestational week during early trimester when the maternal weight was measured, mid-upper arm circumference and randomized regimens, household wealth at enrollment, and infant sex.
