# Supplementary material for: Associations between gestational weight gain under different guidelines and adverse birth outcomes: A secondary analysis of a randomized controlled trial in rural western China
Source: PLOS Glob Public Health. 2024 Jan 8;4(1):e0002691. doi: 10.1371/journal.pgph.0002691 (PMC10773947; doi:10.1371/journal.pgph.0002691)
Supplement: S10 Table — (DOCX) [file pgph.0002691.s010.docx]

S10 Table. E-values for the association between different GWG classifications and adverse birth outcomes among pregnant women with normal weight (n=1,239).

|  | IOM category | | NHC category | | z-score category 1 | | z-score category 2 | |
| --- | --- | --- | --- | --- | --- | --- | --- | --- |
|  | Inadequate | Excessive | Inadequate | Excessive | Below average | Above average | Below average | Above average |
| Preterm birth | 1.21 | 1.11 | 1.37 | 1.25 | 1.34 | 1.32 | 1.62 | 2.17 |
| Post-term birth | 3.70 | 3.27 | 3.78 | 2.79 | 1.39 | 4.85 | 1.71 | 5.91 |
| LBW | 2.15 | 1.64 | 1.53 | 1.63 | 1.51 | 2.45 | 1.54 | 2.52 |
| Macrosomia | 2.71 | 4.64 | 2.34 | 6.94 | 2.45 | 4.01 | 1.32 | 4.72 |
| SGA | 1.81 | 2.06 | 1.76 | 1.69 | 1.11 | 1.31 | 1.17 | 1.59 |
| LGA | 2.41 | 3.93 | 1.62 | 3.60 | 2.55 | 1.17 | 1.96 | 1.97 |

E-values are calculated based on the results displayed in Table 3.

Abbreviations: GWG, gestational weight gain; IOM, Institute of Medicine; NHC, National Health Commission; LBW, low birth weight; SGA, small-for-gestational-age; LGA, large-for-gestational-age.
