# Supplementary material for: Associations between gestational weight gain under different guidelines and adverse birth outcomes: A secondary analysis of a randomized controlled trial in rural western China
Source: PLOS Glob Public Health. 2024 Jan 8;4(1):e0002691. doi: 10.1371/journal.pgph.0002691 (PMC10773947; doi:10.1371/journal.pgph.0002691)
Supplement: Attachment — Submitted filename: PGPH-D-23-00983_reviewer_Comments.pdf [file pgph.0002691.s012.pdf]

PLOS Global Public Health

Associations between gestational weight gain in different guidelines and adverse birth outcomes: a secondary analysis of a randomized controlled trial in rural western China

--Manuscript Draft--

Manuscript Number:	PGPH-D-23-00983
Article Type:	Research Article
Full Title:	Associations between gestational weight gain in different guidelines and adverse birth outcomes: a secondary analysis of a randomized controlled trial in rural western China
Short Title:	gestational weight gain and birth outcomes
Corresponding Author:	Lingxia Zeng, Ph.D<br>Xi'an Jiaotong University Health Science Center<br>Xi'an, Shaanxi Province CHINA
Order of Authors:	Yingze Zhu
	Liang Wang
	Qi Qi
	Yue Cheng
	Zhonghai Zhu
	Lingxia Zeng
Keywords:	gestational weight gain; birth weight; gestational age
Abstract:	Several gestational weight gain (GWG) guidelines have been established based on monocenter or multicenter researches. We aimed to examine the associations between categories of gestational weight gain (GWG) and adverse birth outcome under the Institute of Medicine recommendation (IOM), the Chinese National Health Commission (NHC) guideline, and weight-gain-for-gestational-age z-scores derived from the INTERGROWTH-21st Project. We used data from a prospective birth cohort of infants born to women who participated in an antenatal micronutrient supplementation trial in rural western China between 2002 and 2006. Maternal weekly average GWG during the second and third trimesters was calculated and classified into inadequate, adequate and excessive GWG according to IOM and NHC. Weight-gain-for-gestational-age z-scores derived from the INTERGROWTH-21st Project were grouped into three subgroups using two approaches: z-score percentile<25th, 25th to 75th, >75th and z-score < -1, -1 to 1, >1 SD. Infant birth weight and gestational age were measured using standard approaches. A generalized linear model with binomial family and logit link was applied to estimate the odds ratio (OR) and 95% confidential intervals (CI) for GWG categories and adverse birth outcome. Among 1,239 women with normal BMI (18.5 kg/m2 to 24.0 kg/m2) during early pregnancy, 18.0% and 34.2% was classified as adequate GWG, according to IOM and NHC, respectively. According to NHC, excessive GWG showed a significant association with macrosomia (OR 3.75, 95%CI 1.03, 13.74), large-for-gestation-age (OR 2.12, 95%CI 1.01, 4.45), and inadequate GWG was associated with post-term birth (OR 2.25, 95%CI 1.21, 4.16). Less than half of Chinese women reached optimal GWG by any recommendation guideline. Inappropriate GWG was associated with adverse birth outcomes even among women with normal BMI during early pregnancy. The monitoring and interventions of weight status during pregnancy, especially for the second and third trimesters, is of great public health importance.
Additional Information:	
Question	Response
Financial Disclosure	The funders had no role in study design, data collection and analysis, decision to publish, or preparation of the manuscript

Powered by Editorial Manager® and ProduXion Manager® from Aries Systems Corporation

Enter a financial disclosure statement that describes the sources of funding for the work included in this submission and the role the funder(s) played. This includes grants and any commercial funding of the work or authors.<br><br>This statement will be typeset if the manuscript is accepted for publication.<br><br>*Please review the submission guidelines and the instructions link below for detailed requirements and guidance.*	
**Competing Interests**<br><br>On behalf of all authors, disclose any competing interests that could be perceived to bias this work.<br><br>This statement will be typeset if the manuscript is accepted for publication.<br><br>*Please review the instructions link below and PLOS Global Public Health's competing interests policy to determine what information must be disclosed at submission.*	The authors have declared that no competing interests exist.
**Data Availability**<br><br>Before publication, Authors are required to make fully available and without restriction all data underlying their findings. Please see our PLOS Data Policy page for detailed information on this policy.<br><br>A **Data Availability Statement**, detailing where the data can be accessed, is required at first submission. Insert your Data Availability Statement in the box below.	The datasets used and/or analyzed during the current study are available from the corresponding author on reasonable request.

Powered by Editorial Manager® and ProduXion Manager® from Aries Systems Corporation

Please see the data reporting section of our submission guidelines for instructions on what you need to include in your Data Availability Statement.<br><br>This statement will be typeset if the manuscript is accepted for publication.<br><br>*PLOS allows rare exemptions to address legal and ethical concerns. If you have legal or ethical restrictions, please use the box below to detail these in full sentences for the Journal team to consider.*	

Powered by Editorial Manager® and ProduXion Manager® from Aries Systems Corporation

Manuscript

Click here to access/download;Manuscript;2023.5.24 Manuscript.docx

Associations between gestational weight gain in different guidelines and adverse birth outcomes: a secondary analysis of a randomized controlled trial in rural western China

Yingze Zhu[1], Liang Wang[1], Qi Qi[1], Yue Cheng[2], Zhonghai Zhu[1,3]*, Lingxia Zeng[1,3,4]*

[1]Department of Epidemiology and Biostatistics, School of Public Health, Xi'an Jiaotong University Health Science Center, Xi'an, Shaanxi 710061, P.R. China;

[2]Department of Nutrition and Food Safety Research, School of Public Health, Xi'an Jiaotong University Health Science Center, Xi'an, Shaanxi 710061, P.R. China;

[3]Key Laboratory for Disease Prevention and Control and Health Promotion of Shaanxi Province, China;

[4]Big Data Sharing Platform for Major Disease Prevention and Control and One Health of Shaanxi Province, China

***Corresponding author:**

E-mail: tjzlx@mail.xjtu.edu.cn (ZL) and zhuzhonghai@hotmail.com (ZZ)

Abstract

Several gestational weight gain (GWG) guidelines have been established based on monocenter or multicenter researches. We aimed to examine the associations between categories of gestational weight gain (GWG) and adverse birth outcome under the Institute of Medicine recommendation (IOM), the Chinese National Health Commission (NHC) guideline, and weight-gain-for-gestational-age z-scores derived from the INTERGROWTH-21st Project. We used data from a prospective birth cohort of infants born to women who participated in an antenatal micronutrient supplementation trial in rural western China between 2002 and 2006. Maternal weekly average GWG during the second and third trimesters was calculated and classified into inadequate, adequate and excessive GWG according to IOM and NHC. Weight-gain-for-gestational-age z-scores derived from the INTERGROWTH-21st Project were grouped into three subgroups using two approaches: z-score percentile<25th, 25th to 75th, >75th and z-score < -1, -1 to 1, >1 SD. Infant birth weight and gestational age were measured using standard approaches. A generalized linear model with binomial family and logit link was applied to estimate the odds ratio (OR) and 95% confidential intervals (CI) for GWG categories and adverse birth outcome. Among 1,239 women with normal BMI (18.5 kg/m^2 to 24.0 kg/m^2) during early pregnancy, 18.0% and 34.2% was classified as adequate GWG, according to IOM and NHC, respectively. According to NHC, excessive GWG showed a significant association with macrosomia (OR 3.75, 95%CI 1.03, 13.74), large-for-gestation-age (OR 2.12, 95%CI 1.01, 4.45), and inadequate GWG was associated with post-term birth (OR 2.25, 95%CI 1.21, 4.16). Less than half of Chinese women reached optimal GWG by any recommendation guideline. Inappropriate GWG was associated with adverse birth outcomes even among women with normal BMI during early pregnancy. The monitoring and interventions of

weight status during pregnancy, especially for the second and third trimesters, is of great public health importance.

Introduction

The prevalence of infants born small-for-gestational-age (SGA) in low- and middle-income countries (LMICs) was as high as 41.5% [1], and the highest prevalence of macrosomia in 2013 was 14.9% in Algeria reported [2]. Adverse birth outcomes lay a disadvantaged foundation for later development, such as SGA and preterm birth, with complex genetic and environmental causes [3, 4]. Maternal weight status is a highly predictive factor of fetal growth, which could be considered a proxy of the intrauterine environment [5]. Accordingly, weight gain during pregnancy, which reflects the growth of the fetus, placenta, and uterus, is another independent predictor of fetal development [6]. Mounting evidence suggested that low gestational weight gain (GWG) may benefit obese women but increase the risk of delivering low birth weight infants for underweight women [7, 8]. Therefore, to ensure a healthy fetus, establishing recommendation range for GWG of different body-mass-index (BMI) categories is necessary. The Institute of Medicine (IOM) guideline promulgated in 2009 were the most widely adopted and commonly applied in clinical practice [9]. Moreover, a individual participant data (IPD) meta-analysis revealed that, compared with maternal GWG within the IOM recommendation range (defined as adequate GWG), inadequate GWG showed a 1.94 times higher risk of preterm and 1.52 times higher risk of SGA [10]. Additionally, for excessive GWG, the risk of large-for-gestational-age (LGA) increased (adjusted odds ratio (aOR): 2.00; 95% confidence interval (CI) 1.58, 2.54) and risk of SGA reduced (aOR: 0.66; 95% CI 0.50,0.87).[10]

However, the establishment of IOM was based on US white women and mainly examined in developed settings, where the incidence of overweight and obesity is much higher than those women residing in LMICs [11]. Notably, surveys across continents and ethnicity showed that Asian women have a higher prevalence of insufficient GWG (31%) compared with American women (21%), according to IOM [11]. The percentage of inadequate GWG was even up to 80% in low-income Sub-Saharan countries [12]. Additionally, the incidence of malnutrition, physical status and lifestyle differ a lot during child-bearing age women across countries [13]. Considering the difference in ethnicity, dietary patterns, environmental exposure and other related factors, many countries have built their cohort to explore the optimal GWG ranges for local populations in recent years [14]. Besides, no consensus has been reached on defining optimal GWG among pregnant women. Furthermore, the IOM recommendation was constructed based on the lowest prevalence of the commonly pregnant outcomes from various extensive studies [9].

In 2022, the National Health Commission (NHC) of the People's Republic of China issued a *Recommended standard for weight gain in pregnant women*. This was implemented on October 1st, 2022, and utilized data from a cohort including more than 100,000 pregnant Chinese women [15]. It considered composite maternal and neonatal pregnancy outcomes, including gestational hypertension, gestational diabetes mellitus, anemia, preterm, SGA, LGA, low birth weight (LBW), macrosomia, and cesarean section. Moreover, the Fetal Growth Longitudinal Study of the INTERGROWTH-21st Project described patterns of GWG among healthy, well-nourished, normal weight, and educated women that are compatible with desirable healthy pregnancy outcomes and generated weight-gain-for-gestational age z-score standards. It was conducted in a

multicenter including eight geographically diverse urban regions in Brazil, China, India, Italy, Kenya, Oman, the United Kingdom, and the United States [16]. However, robust epidemiological evidence has not identified the optimal centile range guiding clinical practice across regions. Furthermore, the INTERGROWTH-21st Project only enrolled women with a BMI of 18.50-24.99kg/m^2 in the first trimester of pregnancy.

Other researchers have examined the association between GWG and perinatal outcomes among Chinese women [8, 17], while most of them applied categorical predictors classified by IOM guideline. In recent years, with the improvement of prenatal and postnatal care, studies investigating appropriate GWG recommendations for Chinese females reported their findings [18, 19]. Chen and her colleague compared the differences between the Chinese Nutrition Society and IOM recommendation for GWG in association with children's body composition (aged 4-5 years) [20]. By assessing sensitivity, specificity, and positive and negative predictive values concerning mothers who keep appropriate GWG to estimate offspring nutritional status, generally higher values based on the Chinese Nutrition Society guideline were found compared with IOM. While those studies almost recruited mother-infant pairs in Beijing, Tianjin, Chengdu, and other relatively developed cities in China, limited literature has focused on the rural population suffering from malnutrition and insufficient antenatal care [21]. Likewise, few investigations have examined the appliance of weight-gain-for-gestational age z-score constructed by the INTERGROWTH-21st Project in China.

In the present study, we used data from a cluster-randomized, double-blind trial in rural western China, where populations suffer from relatively disadvantaged socioeconomic status with limited health workforce, health infrastructure and maternal care [22]. Thus, we aimed to examine the association between GWG classifications and adverse birth

outcomes among rural western Chinese mother-infant pairs under the NHC and IOM guidelines separately. In addition, we aimed to explore the association between the weight-gain-for-gestational age z-scores constructed by the INTERGROWTH-21st Project and birth outcomes.

Materials and Methods

Study design and participants

We used data from a cluster-randomized, double-blind trial in rural western China between August 2002 and February 2006 (ISRCTN08850194). This trial primarily aimed to investigate the effect of prenatal micronutrient supplementation on birthweight and neonatal mortality. Briefly, all pregnant women from every village in two counties were eligible to enroll in this trial and were randomized to take a daily capsule of folic acid, iron-folic acid (IFA), or multiple micronutrients (MMN) until delivery. The details of this trial have been clearly described in the report [23]. Notably, singleton live births were included in the final analysis. Ethical approvals for the trial were received from the Ethics Committee of Xi'an Jiaotong University Health Science Center. Furthermore, written informed consent was obtained from mothers.

To focus on the impact of GWG regardless of malnutrition status (underweight and overweight/obesity) in pre-pregnancy, data from pregnant women with normal BMI were included in the final analysis. In addition, due to the difference in BMI cut-offs by IOM and NHC, we restricted the sample size to pregnant women with normal BMI defined by the NHC BMI category (18.5 kg/m^2≤BMI＜24.0 kg/m^2), a narrower criterion.

Measurements

Gestational weight gain

Pregnant women enrolled in the original prenatal trial could receive at least three-time antenatal care, at which trained maternal and child health staff measured their height and weight using standardized equipment and procedures. Maternal BMI during the first trimester (<14 weeks) was used as a proxy for pre-pregnancy BMI in the present study to classify GWG, given that maternal weight changes usually occur after the first trimester [24]. Therefore, we used data from women who enrolled before 14 weeks of gestation.

We calculated weekly gestational weight gain (kg/week) during the second and third trimesters. We selected GWG velocity as a predictor because it is independent of gestational age. According to IOM and NHC guidelines, inadequate, adequate and excessive GWG were defined as below, within, and above the recommended range of GWG velocity during the second and third trimesters [9, 15]. The recommended range by IOM was 0.35-0.50kg/week for women with normal weight, while the NHC recommended 0.26-0.48kg/week for pregnant women classified as normal.

From the Fetal Growth Longitudinal Study of the INTERGROWTH-21st Project standards, we calculated weight-gain-for-gestational age z-score and z-score percentiles for each pregnant woman [16]. However, which cut off points are associated with the best health outcomes is unclear. Therefore, z-scores were grouped into three groups by general rules in two ways: category 1 based on percentiles of z-score (z-score percentile<25th, 25th to 75th, >75th) and category 2 based on origin z-score (< -1, -1 to 1, >1).

Infant birth outcomes

Birth weights were measured within one hour of delivery by hospital nursing staff with an electronic scale (type BD 585, Tanita, Dongguan, Guangdong Province, China) if delivered in the six counties hospital and three largest township hospitals. For other smaller township hospitals and home births, birth weight was measured with a baby scale (type RTZ-10A-RT, Wuxi Weigher Factory, Wuxi, China). Low birth weight was defined as <2500 g, and macrosomia was defined as >4000 g. Gestational age at birth was measured as completed days based on the first day of the last menstrual period obtained at the baseline interview. Preterm delivery was defined as delivery before 37 weeks, and post-term birth was defined as delivery after 42 weeks. SGA and LGA babies were defined as having a birth weight of less than the 10th or more than the 90th percentile for gestational age (INTERGROWTH-21st Project) [25].

Other covariables

Covariables were collected by face-to-face interviews following standard procedures. We included parental age in years, education attainment level (did not complete primary school, primary school, secondary school, high school diploma or greater), occupation (farmer, others), household wealth, maternal mid-upper arm circumference at the enrollment (continuous variable), parity (0, 1, ≥2) , randomized regimens (folic acid, folic acid plus iron and multiple micronutrients), and pre-pregnancy medical history (including cardiac disease, hypertension, diabetes, tuberculosis, kidney disease, chronic liver disease, hyperthyroidism and or anemia). The household wealth index at the enrollment was constructed using principal component analysis for household assets and dwelling characteristics.

Statistical analysis

Continuous variables were presented as mean and standard deviations and categorical variables were expressed as numbers (%, percentage). The Kappa test was used to compare GWG classifications by different approaches. To examine the association between GWG category and preterm delivery, post-term delivery, LBW, macrosomia, SGA, and LGA, a generalized linear model with binomial family and logit link was applied, with adequate GWG or middle subgroup set as reference. The areas under the curve (AUC) for adverse birth outcomes predicted by each cut-off value were also calculated. In addition, birthweight for gestational age z-score and gestational age at birth was treated as continuous outcomes fitted with a generalized linear model. The association between continuous weekly average GWG and weight-gain-for-gestational-age z-scores derived from INTERGROWTH-21st Project and birth outcomes (both dichotomous and continuous variables) were also examined. We considered the following potential confounders in the model: parental education, occupation and age, maternal parity, the gestational week during the first trimester, mid-upper arm circumference, randomized regimens and past medical history, household wealth at enrollment and infant sex.

For supplementary analyses, we applied E-value to assess the potential effect of unmeasured confounders [26]. Besides, we examined the association between categorial predictors and adverse outcomes among mother-infant pairs with abnormal BMI classified by IOM and NHC recommendations. To explore if the association was modified by infant sex or parental education level, the interaction term was added to the model to assess the statistical significance.

All *P* values were 2-sided with an alpha of 0.05. Statistical analyses were conducted using Stata 15.0 (Stata Corp, College Station, Texas, USA)

Results

A total of 1,239 mother-infant pairs were included in the final analysis. As shown in Table 1, the prevalence of preterm, post-term birth, LBW, macrosomia, SGA, and LGA was 3.7%, 5.8%, 3.9%, 1.5%, 13.6%, and 4.9%, respectively. Table 2 shows that most women did not achieve the recommended range for gestational weight gains defined by each classification. The proportion of women who achieved optimal GWG rates during the second and third trimesters under IOM and NHC guideline was 18.0% and 34.2%, respectively. More women were classified as inadequate GWG based on IOM guideline compared with NHC guideline (64.4% versus 46.7%). Additionally, the Kappa value between IOM and NHC recommendation was 0.68. For categorical groups of weight-gain-for-gestational-age z-scores derived from the INTERGROWTH-21st Project, the proportion of the middle group was 28.4% (z-score percentiles in 25th to 75th) and 39.8% (z-score in -1 to 1).

Table 1. Characteristics of the pregnant women and their offspring (n=1,239).

**Factors**	**No. (%)**
Pregnant women characteristics	
**Maternal age (years) /Mean (SD)**	24.5 (4.5)
**Maternal education**	
**< 3 years**	63 (5.1)
**Primary**	301 (24.4)
**Secondary**	670 (54.2)
**High school and above**	202 (16.34)
**Maternal occupation**	
**Farmer**	1000 (81.23)
**Others**	231 (18.7)

**Parity at enrollment**	
**0**	817 (65.9)
**1**	378 (30.5)
**≥2**	44 (3.5)
**Maternal MUAC (cm)**	
**<21.5**	171 (13.9)
**≥21.5**	1062 (86.13)
**Randomized regimens**	
**Folic acid**	456 (36.8)
**Folic acid plus iron**	421 (34.0)
**Multiple micronutrient**	362 (29.2)
**Household wealth at enrollment[a]**	
**Low**	
**Medium**	376 (30.35)
**High**	418 (33.74)
Infant characteristics	445 (35.92)
**Sex**	
**Male**	693 (55.9)
**Female**	546 (44.1)
**Birth weight (gram) /Mean (SD)**	3189 (411)
**Birthweight for gestational age z-score/Mean (SD)**	-0.24 (0.99)
**Gestational age at birth/Mean (SD)**	39.8 (1.5)
**Preterm birth (< 37 gestational weeks)**	46 (3.7)
**Post-term birth(≥42 gestational weeks)**	72 (5.8)
**Low birth weight (< 2500 g)**	48 (3.9)
**Macrosomia(>4000 g)**	19 (1.5)
**Small-for-gestational age (<10th percentile)**	169 (13.6)
**Large-for-gestational age (>90th percentile)**	61 (4.9)

Abbreviations: SD: standard deviations; MUAC, mid-upper arm circumference.

[a]Household wealth at enrollment was derived from principal component analysis for household assets and dwelling characteristics, and was further categorized by its terciles.

Table 2. Classification of weekly average gestational weight gain during the second and third trimesters and consistency among different guideline (n=1,239).

**Measure**	**GWG categories [n(%)]**			**Kappa**		
	**Below[a]**	**Within[a]**	**Above[a]**			
**IOM**	798 (64.4)	223 (18.0)	218 (17.6)	Ref.		
**NHC**	579 (46.7)	424 (34.2)	236 (19.1)	0.68	Ref.	
**Z-score cat1[b]**	804 (64.9)	352 (28.4)	83 (6.7)	0.47	0.34	Ref.
**Z-score cat2[c]**	689 (55.6)	493 (39.8)	57 (4.6)	0.45	0.43	0.78

Abbreviations: GWG, gestational weight gain; IOM, Institute of Medicine; NHC, National Health Commission.

[a]Below, within, and above classification were defined as below, within, and above the recommended range defined by IOM or NHC. For gestational-weight-gain-for-gestation-age z-scores derived from INTERGROWTH-21st Project, below, within, and above refer to subgroups 1,2,3, respectively.

[b]Z-score category 1 refers to subjects classified into three groups by percentiles of gestational-weight-gain-for-gestation-age z-score derived from INTERGROWTH-21st Project (z-score percentile<25th, 25th to 75th, >75th).

[c]Z-score category 2 refers to subjects classified into three groups by of gestational-weight-gain-for-gestation-age z-score derived from INTERGROWTH-21st Project (z-score< -1, -1 to 1, >1).

Table 3 shows the association between different GWG classifications and adverse birth outcomes, and statistical associations with several outcomes were only observed for the NHC category. We found that inadequate GWG contributed to a 2.25 (95%CI 1.21, 4.16) times higher risk for post-term birth. In comparison, excessive GWG contributed to a 3.75 (95%CI 1.03, 13.74) times higher risk for macrosomia and a 2.12 (95%CI 1.01, 4.45) times higher risk for LGA compared with adequate GWG. However, we observed no significant association between GWG classifications with birth outcomes which were analyzed as continuous variables, including birth weight, birthweight for gestational age z-score, and gestational age at birth (see S1 Table). The AUC of different birth outcomes predicted by each cut-off value was between 0.6162 and 0.8058 and showed no significant difference, as shown in Fig 1 and S2 Table.

Fig 1. The areas under the curve (AUC) for adverse birth outcomes predicted by each cut-off values of GWG. Abbreviations: GWG, gestational weight gain; IOM, Institute of Medicine; NHC, National Health Commission. Z-score category 1 refers to subjects classified into three groups by percentiles of z-score (z-score percentile<25th, 25th to 75th, >75th). We set subgroup 2 (25th to 75th) as the reference

group. Z-score category 2 refers to subjects classified into three groups by of z-score (< -1, -1 to 1, >1). We set subgroup 2 (-1 to 1) as reference group.

Continuous GWG variables were also tested, including weekly average weight gains during the second and third trimesters and gestational weight-gain-for-gestational-age z-score derived from the INTERGROWTH-21st Project. We performed similar analyses as before and observed a positive association between z-score and birth weight (mean difference (MD) 21.57g; 95%CI 3.48, 39.67), birthweight for gestational age z-score (MD 0.05; 95%CI 0.01, 0.10), and LGA (OR 1.28; 95%CI 1.01, 1.61) (see S3 Table). However, for z-score groups classified by general rules in two ways (z-score percentile<25th, 25th to 75th, >75th and z-score <-1, -1 to 1, >1), no significant association was observed between subgroups and birth outcomes (both continuous outcomes and dichotomous outcomes). In addition, by including all pregnant women with thinness or overweight/obesity in the analyses, the classifications based on IOM or NHC were shown in S4 Table The association between categorical predictors and adverse outcomes is shown in S5 Table. Notably, we observed a similar association between the NHC category and post-term birth, macrosomia, and LGA. All p values for the interaction term between GWG category, parental educational attainment level, and infant sex were >0.05 (see S6 Table). Furthermore, E-values for associations between inappropriate GWG and post-term birth, macrosomia, and LGA showed relatively robust (see S7 Table).

Table 3. Association between different GWG classifications and adverse birth outcomes among pregnant women with normal weight (n=1,239).

	IOM category[a]		NHC category[a]		z-score category 1[ab]		z-score category 2[ac]	
	Inadequate	Excessive	Inadequate	Excessive	z-score percentile<25th	z-score percentile>75th	z-score < -1	z-score>1
**Preterm birth**	0.81 (0.35, 1.86)	0.98 (0.35, 2.70)	0.93 (0.46, 1.89)	1.00 (0.41, 2.43)	0.96 (0.48, 1.95)	0.99 (0.26, 3.71)	1.04 (0.54, 2.03)	1.48 (0.40, 5.45)
**Post-term birth**	2.09 (0.92, 4.72)	1.77 (0.68, 4.64)	2.25 (1.21, 4.16)	1.64 (0.76, 3.55)	0.93 (0.54, 1.60)	0.42 (0.09, 1.82)	1.23 (0.73, 2.07)	0.36 (0.05, 2.76)
**LBW**	1.25 (0.50, 3.09)	1.08 (0.35, 3.35)	1.03 (0.52, 2.02)	0.80 (0.31, 2.04)	1.10 (0.54, 2.27)	1.52 (0.45, 5.11)	1.12 (0.58, 2.17)	1.49 (0.40, 5.55)
**Macrosomia**	1.83 (0.39, 8.57)	3.00 (0.55, 16.23)	1.55 (0.44, 5.43)	3.75 (1.03, 13.74)	0.66 (0.23, 1.93)	2.13 (0.48, 9.33)	0.97 (0.35, 2.69)	2.42 (0.45, 12.92)
**SGA**	1.22 (0.75, 1.97)	1.31 (0.73, 2.35)	1.22 (0.83, 1.80)	1.17 (0.72, 1.90)	1.01 (0.68, 1.48)	1.12 (0.55, 2.28)	1.03 (0.72, 1.48)	1.30 (0.59, 2.85)
**LGA**	1.37 (0.58, 3.25)	2.18 (0.84, 5.64)	1.10 (0.55, 2.20)	2.12 (1.01, 4.45)	0.57 (0.31, 1.06)	1.01 (0.37, 2.78)	0.71 (0.39, 1.30)	1.42 (0.48, 4.21)

Abbreviations: GWG, gestational weight gain; IOM, Institute of Medicine; NHC, National Health Commission; LBW, low birth weight; SGA, small-for-gestational-age; LGA, large-for-gestational-age.

[a]Data are presented with adjusted odd ratios and 95% confidence intervals by performing generalized linear models. The adjustments included parental education, occupation and age, maternal parity, gestational week during first trimester, mid-upper arm circumference, randomized regimens and past medical history, household wealth at enrollment, and infant sex.

[b]Z-score category 1 refers to subjects classified into three groups by percentiles of z-score (z-score percentile<25th, 25th to 75th, >75th). We set subgroup 2 (25th to 75th) as the reference group.

[c]Z-score category 2 refers to subjects classified into three groups by of z-score (< -1, -1 to 1, >1). We set subgroup 2 (-1 to 1) as the reference group.

Discussion

This study found that half of the pregnant women in rural western China gained gestational weight below the recommendation under each standard. Only 18.0% and 34.2% of pregnant women reached adequate gestational weight gains rate during the second and third trimesters identified by the IOM guideline and Chinese 2022 guideline, respectively. In addition, our results suggested that inadequate weekly weight gains during the second and third trimesters were associated with higher risk of post-term birth (OR 2.25; 95%CI 1.21, 4.16) and excessive GWG raised the risk of macrosomia (OR 3.75; 95%CI 1.03, 13.74) and a LGA (OR 2.12; 95%CI 1.01, 4.45).

The key question of GWG recommendation is whether optimal weight gains can improve maternal and offspring outcomes. Both total weight gains during entire pregnancy and incremental rate need to be emphasized [27]. Likewise, population and regional differences contribute to various women's body sizes and weight gain profiles [11]. In addition, regional socioeconomic, medical, and cultural factors also play a role [28]. Therefore, developing their own BMI classification criteria has been emphasized based on their own morbidity and mortality data [29], for the recommended GWG range. Several studies explored their own optimal GWG ranges suitable and sensible for regional pregnant women by various approaches. Large epidemiological studies are needed to identify those recommendation ranges to applying to clinical guidance. In our study, by testing three GWG standards, we found that for post-term birth, macrosomia, and LGA, outcomes are associated with suboptimal GWG categories classified by NHC guideline. In contrast, models based on IOM and z-scores categories did not yield similar patterns of ORs. For continuous birth outcomes variables, birth weight and

birthweight for gestational-age z-score were positively linked with gestational-weight-gains-for gestational-age z-score derived from INTERGROWTH-21st Project.

Previous studies have shown that the risk of post-term birth increase with increasing maternal BMI [30]. At the same time, we found that inadequate GWG was associated with a higher risk of post-term birth among mothers with normal BMI. Insufficient maternal energy intake may be linked causally with a greater risk of fetal growth restriction [31], prolonging the gestational week. We overthrew the systematic review and meta-analyses, which focus on GWG and infant outcomes, and few pieces of evidence mentioned post-term birth [6, 32, 33]. However, even in low-risk singleton pregnancies, post-term pregnancy appears to be an independent risk factor for adverse neonatal outcomes, including respiratory morbidity, infectious morbidity and hypoglycemia [34]. Additionally, it might have a lasting impact by raising the risk of developmental vulnerability through-out early childhood [35–37]. Therefore, preterm and post-term should be considered as an important neonatal outcome and explore related risk factors in further studies.

A meta-analysis including 1,309,136 women revealed that GWG above the recommendations showed a higher risk of LGA (OR 1.85; 95%CI 1.76, 1.95) and macrosomia (OR 1.95; 95%CI 1.79, 2.11). However, most studies were conducted in developed settings [6]. A study among American Samoan women found that third-trimester GWG was positively linked to the risk of a LGA baby among normal and underweight women (hazard ratio (HR) 3.21; 95%CI 1.50, 6.89), which reflects the period of in-utero weight and fat gain for fetus [27]. However, the results remain inconsistent in LMICs which might be due to the high prevalence of malnutrition. A

systematic review of studies conducted in sub-Saharan Africa reported that among seven of the sixteen studies, the percentages of women who gained excessive gestational weight were<10%. No significant association was observed between excessive GWG and macrosomia [12]. However, more than half of those studies were based on low-income settings with various health challenges [12]. In the present study, conducted in rural western China between 2002 and 2006, we observed that mothers who gained weekly average gestational weight during second and third trimesters above the recommendation range defined by NHC showed a greater risk of delivering macrosomia and LGA infants even among pregnant women with normal BMI. Notably, these results emphasize the importance of weight management during the whole pregnancy period, especially for the second and third trimesters, to avoid excessive GWG regardless of pre-pregnancy nutrition status.

Previous studies have highlighted the importance of monitoring the weight status among women pre-pregnancy and during pregnancy [31]. Besides, Gondwe and his colleague reported a discrepancy between underweight (5.9%) and low GWG (71.8%), suggesting that factors other than pre-pregnancy nutrition status contribute to suboptimal GWG [38]. In the present study, we focused on pregnant women with residing in rural western China with normal BMI. Under the NHC guideline, 46.7% of pregnant women failed to reach the recommended GWG range which might due to poor resources and limited antenatal care. Additionally, the proportion of excessive GWG was up to 19.1% and linked with macrosomia and LGA. Moreover, the definition of normal weight is narrower based on Chinese-specific BMI standards than IOM recommendation which is consistent with WHO standards, suggesting fewer women were classified as normal weight. Therefore, previous studies which grouped pre-

pregnancy BMI according to WHO standards would underestimate the hazards of pre-pregnancy overweight and excessive GWG. Moreover, compared to estimating total weight gains, we used approaches of GWG rate to account for the bias caused by gestational age [39]. The evidence showed that the second and third trimester was the maximal growth and weight gain for fetuses and were significantly associated with newborn weight [40]. Therefore, we calculated the weekly average GWG during the second and third trimesters as the indicator. Besides, the z-scores derived from INTERGROWTH-21st Project was grouped by two general rules: z-score percentile<25th, 25th to 75th, >75th and z-score< -1, -1 to 1, >1. However, no significant association was observed between subgroups and birth outcomes, possibly due to the inappropriate classification approaches. Thus, epidemiological studies with large sample data are needed to identify optimal centiles in further research.

Several limitations that should be noted. First, the mother-infant pairs in our cohort study derived from the origin prenatal cluster-randomized controlled trial, in which pregnant women received micronutrient supplementation (folic acid, folic acid plus iron, and multiple micronutrients), so the generalization of the results might be limited. A study using IPD in LMICs concluded that maternal MMN supplementation was associated with greater GWG proportion and total GWG at delivery compared with folic acid plus iron only [41]. The Bauserman team conducted a mediation analysis to test whether GWG was a mediator between the treatment arm and birth length and weight [42]. In their primary trial, women were randomly assigned lipid-based micronutrient supplementation at preconception (Arm 1), end of the first trimester (Arm 2) and no supplementation (Arm 3), respectively. They reported a significant mediation effect between Arm 2 and 3 concerning birth length (coefficient 0.021; 95%CI 0.003,

0.047) and weight (coefficient 0.006; 95%CI 0.001, 0.012). The present study observed similar GWG velocity and z-scores generated from INTERGROWTH 21st Project by treatment arms (see S8 Table). We recognized that GWG is on the causal pathway between nutrition supplementation and fetal growth and could mediate and/or modify the relation. Still, we failed to elucidate it thoroughly in our study. Secondly, classifying GWG by maternal BMI during the first trimester instead of pre-pregnancy in the guidelines may result in misclassification to some extent, while we primarily aimed to compare results across different methods commonly implemented in the literature. All these methods would be similarly affected by this misclassification. Besides, we also adjust the gestational age in the first trimester. Thirdly, pregnancy complications were associated with GWG by influencing diet patterns, physical activity, and other lifestyles once diagnosed definitely, such as gestational diabetes mellitus [43]. Although we failed to take pregnant complications as confounders into models in the present study, pre-pregnant disease history including hypertension, diabetes, heart disease, and anemia was considered. Furthermore, we applied E-values to assess unmeasured confounders, which showed the robustness of our results.

Conclusions

Our findings suggest that more than half of pregnant women were classified as having suboptimal gestational weight gain according to the either of recommendations in rural western China among women with normal BMI. However, a significant association was only observed in suboptimal GWG and adverse outcomes according to the Chinese National Health Commission guideline promulgated in 2022. Therefore, monitoring the weight status of Chinese women during pregnancy under the proper range

recommended by the Chinese National Health Commission could decrease in the risk of post-term birth, macrosomia, and large-for-gestational-age.

Acknowledgments

We thank all field workers who helped with data collection. We are also grateful to all participants and their families. The authors thank AiMi Academic Services (www.aimieditor.com) for English language editing and review services.

Reference

1 Lee ACC, Katz J, Blencowe H, Cousens S, Kozuki N, Vogel JP, et al. National and regional estimates of term and preterm babies born small for gestational age in 138 low-income and middle-income countries in 2010. Lancet Glob Health. 2013;1:e26-362

2 Koyanagi A, Zhang J, Dagvadorj A, Hirayama F, Shibuya K, Souza JP, et al. Macrosomia in 23 developing countries: an analysis of a multicountry, facility-based, cross-sectional survey. Lancet Lond Engl 2013;381:476-83.

3 Humberg A, Fortmann I, Siller B, Kopp MV, Herting E, Göpel W, et al. Preterm birth and sustained inflammation: consequences for the neonate. Semin Immunopathol 2020;42:451-68.

4 Sacchi C, Marino C, Nosarti C, Vieno A, Visentin S, Simonelli A. Association of intrauterine growth restriction and small for gestational age status with childhood cognitive outcomes: a systematic review and meta-analysis. JAMA Pediatr 2020;174:772-81.

5 Lawlor DA, Smith GD, O'Callaghan M, Alati R, Mamun AA, Williams GM, et al. Epidemiologic evidence for the fetal overnutrition hypothesis: findings from the

mater-university study of pregnancy and its outcomes. Am J Epidemiol 2007;165:418-4.

6 Goldstein RF, Abell SK, Ranasinha S, Misso M, Boyle JA, Black MH, et al. Association of gestational weight gain with maternal and infant outcomes: a systematic review and meta-analysis. JAMA 2017;317:2207-5.

7 Nohr EA, Vaeth M, Baker JL, Sørensen TI, Olsen J, Rasmussen KM. Combined associations of prepregnancy body mass index and gestational weight gain with the outcome of pregnancy. Am J Clin Nutr 2008;87:1750-9.

8 Wang Y, Ma H, Feng Y, Zhan Y, Wu S, Cai S, et al. Association among pre-pregnancy body mass index, gestational weight gain and neonatal birth weight: a prospective cohort study in China. BMC Pregnancy Childbirth 2020;20:690.

9 Rasmussen KM, Yaktine AL, Institute of Medicine (U.S.), editors. Weight gain during pregnancy: reexamining the guidelines. Washington, DC: National Academies Press; 2009.

10 Rogozińska E, Zamora J, Marlin N, Betrán AP, Astrup A, Bogaerts A, et al. Gestational weight gain outside the Institute of Medicine recommendations and adverse pregnancy outcomes: analysis using individual participant data from randomised trials. BMC Pregnancy Childbirth 2019;19:322.

11 Goldstein RF, Abell SK, Ranasinha S, Misso ML, Boyle JA, Harrison CL, et al. Gestational weight gain across continents and ethnicity: systematic review and meta-analysis of maternal and infant outcomes in more than one million women. BMC Med 2018;16:153.

12 Asefa F, Cummins A, Dessie Y, Hayen A, Foureur M. Gestational weight gain and its effect on birth outcomes in sub-Saharan Africa: Systematic review and meta-analysis. PloS One 2020;15:e0231889.

13 Barankanira E, Iradukunda A, Ntakaburimvo N, Ahishakiye W, Nsavyimana JC, Odjidja EN. Spatial distribution and predictive factors of antenatal care in Burundi: A spatial and multilevel baseline analysis for the third Burundian demographic and health survey. PloS One 2023;18:e0272897.

14 Callaghan S, O'Brien E, Coughlan B, McAuliffe FM. Midwives' and obstetricians' level of knowledge of appropriate gestational weight gain recommendations for pregnancy: A systematic review. Birth Berkeley Calif 2020;47:322–31.

15 Standard of recommendation for weight gain during pregnancy period: WS/T 801-2022. 2022. http://www.nhc.gov.cn/fzs/s7848/202208/2c1c388fcd0c47c58630e5f971ebb468.shtml Accessed July 28,2022.

16 Cheikh Ismail L, Bishop DC, Pang R, Ohuma EO, Kac G, Abrams B, et al. Gestational weight gain standards based on women enrolled in the Fetal Growth Longitudinal Study of the INTERGROWTH-21st Project: a prospective longitudinal cohort study. BMJ 2016;352:i555.

17 Huang A, Ji Z, Zhao W, Hu H, Yang Q, Chen D. Rate of gestational weight gain and preterm birth in relation to prepregnancy body mass indices and trimester: a follow-up study in China. Reprod Health 2016;13:93.

18 Sun Y, Shen Z, Zhan Y, Wang Y, Ma S, Zhang S, et al. Investigation of optimal gestational weight gain based on the occurrence of adverse pregnancy outcomes for Chinese women: a prospective cohort study. Reprod Biol Endocrinol 2021;19:130.

19 Zhang CX, Lai JQ, Liu KY, Yang NH, Zeng G, Mao LM, et al. Optimal gestational weight gain in Chinese pregnant women by Chinese-specific BMI categories: a multicentre prospective cohort study. Public Health Nutr 2021;24:3210-20.

20 Chen F, Wang P, Wang J, Liao Z, Zong X, Chen Y, et al. Analysis and comparison of early childhood nutritional outcomes among offspring of Chinese women under the Chinese 2021 and us 2009 gestational weight gain guidelines. JAMA Netw Open 2022;5:e2233250.

21 Huang Y, Shallcross D, Pi L, Tian F, Pan J, Ronsmans C. Ethnicity and maternal and child health outcomes and service coverage in western China: a systematic review and meta-analysis. Lancet Glob Health 2018;6:e39-56.

22 Gao Y, Zhou H, Singh NS, Powell-Jackson T, Nash S, Yang M, et al. Progress and challenges in maternal health in western China: a Countdown to 2015 national case study. Lancet Glob Health 2017;5:e523-36.

23 Zeng L, Cheng Y, Dang S, Yan H, Dibley MJ, Chang S, et al. Impact of micronutrient supplementation during pregnancy on birth weight, duration of gestation, and perinatal mortality in rural western China: double blind cluster randomised controlled trial. BMJ 2008;337:a2001. doi:10.1136/bmj.a2001

24 Fattah C, Farah N, Barry SC, O’Connor N, Stuart B, Turner MJ. Maternal weight and body composition in the first trimester of pregnancy. Acta Obstet Gynecol Scand 2010;89:952-5.

25 Grantz KL, Hediger ML, Liu D, Buck Louis GM. Fetal growth standards: the NICHD fetal growth study approach in context with INTERGROWTH-21st and the World Health Organization Multicentre Growth Reference Study. Am J Obstet Gynecol 2018;218:S641-S655.e28.

26 VanderWeele TJ, Ding P. Sensitivity Analysis in Observational Research: Introducing the E-Value. Ann Intern Med 2017;167:268.

27 Darling AM, Werler MM, Cantonwine DE, Fawzi WW, McElrath TF. Timing and amount of gestational weight gain in association with adverse birth outcomes. Epidemiol Camb Mass 2019;30:695-705.

28 Drewnowski A, Moudon AV, Jiao J, Aggarwal A, Charreire H, Chaix B. Food environment and socioeconomic status influence obesity rates in Seattle and in Paris. Int J Obes 2005 2014;38:306-14.

29 WHO Expert Consultation. Appropriate body-mass index for Asian populations and its implications for policy and intervention strategies. Lancet Lond Engl 2004;363:157-63.

30 Heslehurst N, Vieira R, Hayes L, Crowe L, Jones D, Robalino S, et al. Maternal body mass index and post-term birth: a systematic review and meta-analysis. Obes Rev 2017;18:293-308.

31 Champion ML, Harper LM. Gestational weight gain: update on outcomes and interventions. Curr Diab Rep 2020;20:11.

32 LifeCycle Project-Maternal Obesity and Childhood Outcomes Study Group, Voerman E, Santos S, Inskip H, Amiano P, Barros H, et al. Association of gestational weight gain with adverse maternal and infant outcomes. JAMA 2019;321:1702-15.

33 Perumal N, Wang D, Darling AM, Wang M, Liu E, Urassa W, et al. Associations between gestational weight gain adequacy and neonatal outcomes in Tanzania. Ann Nutr Metab 2022;78:156-65.

34 Linder N, Hiersch L, Fridman E, Klinger G, Lubin D, Kouadio F, et al. Post-term pregnancy is an independent risk factor for neonatal morbidity even in low-risk singleton pregnancies. Arch Dis Child Fetal Neonatal Ed 2017;102:F286-90.

35 Smithers LG, Searle AK, Chittleborough CR, Scheil W, Brinkman SA, Lynch JW. A whole-of-population study of term and post-term gestational age at birth and children’s development. BJOG Int J Obstet Gynaecol 2015;122:1303-11.

36 Abel K, Heuvelman H, Wicks S, Rai D, Emsley R, Gardner R, et al. Gestational age at birth and academic performance: population-based cohort study. Int J Epidemiol 2017;46:324–35.

37 El Marroun H, Zeegers M, Steegers EAP, van der Ende J, Schenk JJ, Hofman A, et al. Post-term birth and the risk of behavioural and emotional problems in early childhood. Int J Epidemiol 2012;41:773-81.

38 Gondwe A, Ashorn P, Ashorn U, Dewey KG, Maleta K, Nkhoma M, et al. Pre-pregnancy body mass index (BMI) and maternal gestational weight gain are positively associated with birth outcomes in rural Malawi. PloS One 2018;13:e0206035.

39 Richards M, Strickland MJ, Flanders WD, Klein M, Darrow LA. Gestational weight gain and birth outcome: a comparison of methods of accounting for gestational age. Am J Epidemiol 2022;191:1687-99.

40 Karachaliou M, Georgiou V, Roumeliotaki T, Chalkiadaki G, Daraki V, Koinaki S, et al. Association of trimester-specific gestational weight gain with fetal growth, offspring obesity, and cardiometabolic traits in early childhood. Am J Obstet Gynecol 2015;212:502.e1-14.

41 Liu E, Wang D, Darling AM, Perumal N, Wang M, Ahmed T, et al. Effects of prenatal nutritional supplements on gestational weight gain in low- and middle-income countries: a meta-analysis of individual participant data. Am J Clin Nutr 2022;116:1864-76.

42 Bauserman MS, Bann CM, Hambidge KM, Garces AL, Figueroa L, Westcott JL, et al. Gestational weight gain in 4 low- and middle-income countries and associations with birth outcomes: a secondary analysis of the Women First Trial. Am J Clin Nutr 2021;114:804-12.
43 Chen CN, Chen HS, Hsu HC. Maternal prepregnancy body mass index, gestational weight gain, and risk of adverse perinatal outcomes in Taiwan: a population-based birth cohort study. Int J Environ Res Public Health 2020;17:e1221.

Supporting information

S1 Table. Association between different GWG classifications and birth weight and gestational age among pregnant women with normal BMI based on Chinese guidelines.

S2 Table. AUC for gestational weight gain recommendation range corresponding to each adverse birth outcome, under IOM, NHC, and z-score, respectively.

S3 Table. Association between different GWG and birth outcomes among pregnant women with normal BMI based on Chinese guidelines.

S4 Table. Classification of weekly average gestational weight gain during the second and third trimesters and consistency among different guidelines based on complete data.

S5 Table. Association between different GWG classifications and adverse birth outcomes among women with underweight, normal, overweight and obese BMI.

S6 Table. Interaction P values between GWG categories and parental education and infant sex for adverse birth outcomes.

S7 Table. E-values for the association between different GWG classifications and adverse birth outcomes among pregnant women with normal BMI.

S8 Table. Average weekly GWG during 2nd and 3rd trimesters and weight-gain-for-gestational-age z scores derived from the INTERGROWTH-21st Project among treatment groups.

Figure
Click here to access/download;Figure;Figure 1.tif

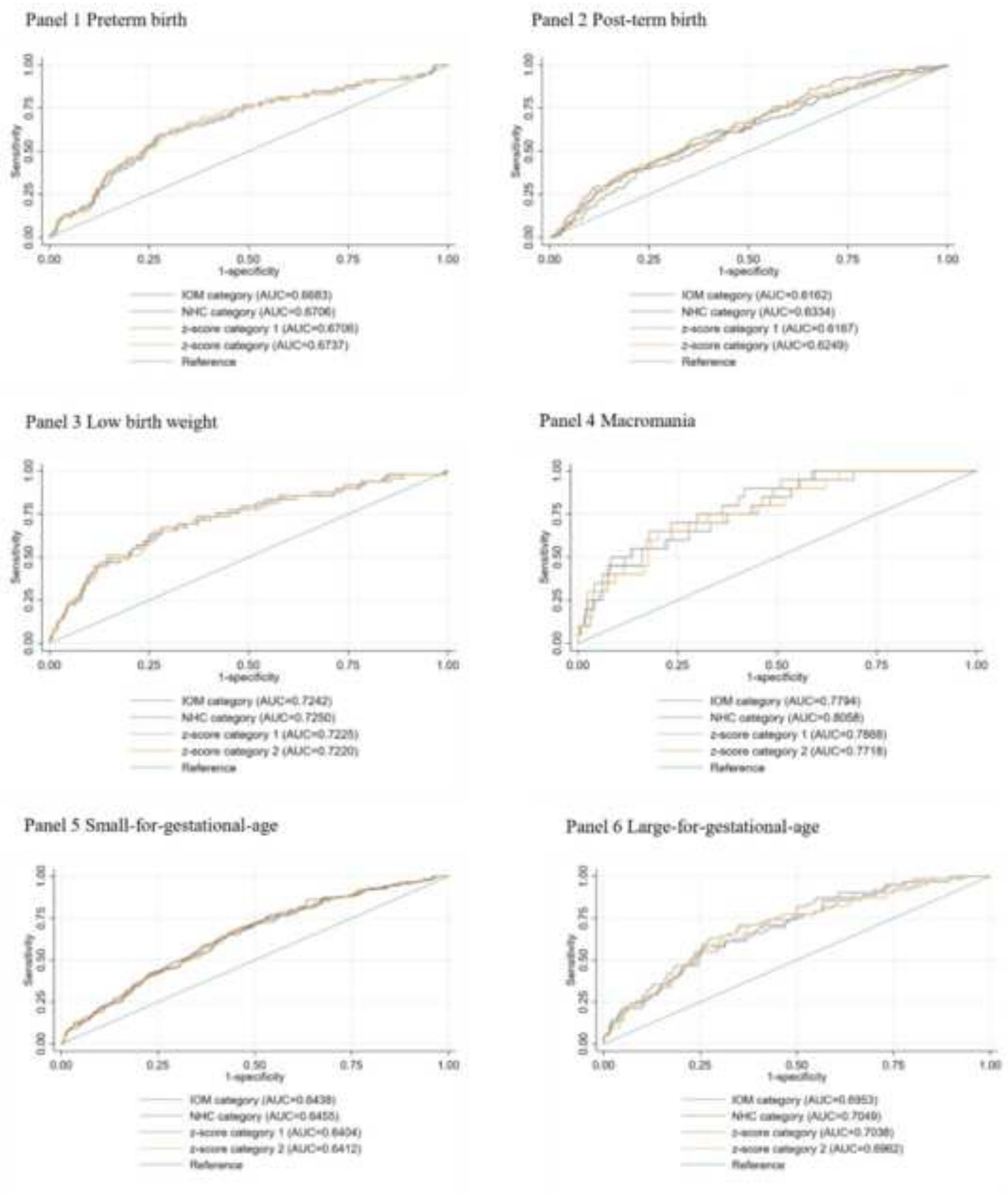

S1 Table

Click here to access/download
Supporting Information
S1_Table.doc.docx

S2Table

Click here to access/download
Supporting Information
S2_Table.doc.docx

S3 Table

Click here to access/download
Supporting Information
S3_Table.doc.docx

S4 Table

Click here to access/download
Supporting Information
S4_Table.doc.docx

S5 Table

Click here to access/download
Supporting Information
S5_Table.doc.docx

S6 Table

Click here to access/download
Supporting Information
S6_Table.doc.docx

S7 Table

Click here to access/download
Supporting Information
S7_Table.doc.docx

S8 Table

Click here to access/download
Supporting Information
S8_Table.doc.docx
