# Supplementary material for: Associations between gestational weight gain under different guidelines and adverse birth outcomes: A secondary analysis of a randomized controlled trial in rural western China
Source: PLOS Glob Public Health. 2024 Jan 8;4(1):e0002691. doi: 10.1371/journal.pgph.0002691 (PMC10773947; doi:10.1371/journal.pgph.0002691)
Supplement: Attachment — Submitted filename: Response to Reviewers.docx [file pgph.0002691.s013.docx]

Detailed Point-to-Point Response to Editors and Reviewers

Manuscript Number: PGPH-D-23-00983

Title: Associations between gestational weight gain in different guidelines and adverse birth outcomes: a secondary analysis of a randomized controlled trial in rural western China

We heartily thank the editors and reviewers for the thoughtful comments and suggestions, which have helped to improve the quality of this manuscript. We appreciate it.

Reviewer #1: This study found a big data to discuss about the different gestational weight gain recommendation in the western chinese women population. the length of discussion need to be divided into several sub-topics. the presentation data need to be improved with some of data present in figure. all comments are attached in the pdf file.

#1. Line 51 Please further which adverse birth outcomes.

*Response*: Thank you for the comment. We have now revised the sentence in detail.

*Lines 51-52 “Inappropriate GWG was associated with adverse birth outcomes macrosomia, LGA, and post-term birth even among women with normal BMI during early pregnancy.”*

#2. Lines 58-59 Please also provide the local community data as well when the study was began.

*Response*: Thank you for the comment. We have now added adverse birth outcomes data in China.

*Lines 86-88 “In China, data from perinatal health care surveillance system in 12 cities showed that the rate of macrosomia rose from 6.00% in 1994 to 8.49% in 2000.”*

#3. Lines 86-87 please provide the evidence from the local community in China

*Response*: Thank you for the comment. We have now added gestational weight gain data in China.

*Lines 108-109 “A recently study conducted in China observed that only 37.2% (1180/3170) women were identified as appropriate GWG according to IOM.”*

#4.Lines 123-124 Is this study will be focused in the rural setting?

*Response*: Thank you for the comment. This study focused on rural settings. The original trial took place in two poor rural counties in Shaanxi Province of north western China. We elaborated the characteristics of study setting in subsequent content.

*Lines 152-154 “In the present study, we used data from a cluster-randomized, double-blind trial in rural western China, where populations suffer from relatively disadvantaged socioeconomic status with limited health workforce, health infrastructure and maternal care.”*

#5. Lines 146-148 Please provide the letter number in bracket following this sentence.

*Response*: Thank you for the comment. We have now added the letter number in bracket following this sentence.

*Lines 149-150 “Ethical approvals for the trial were received from the Ethics Committee of Xi’an Jiaotong University Health Science Center (No 2002001).”*

#6. Lines 167-168 why authors did not included the first trimester?

*Response*: Thank you for the comment. Evidence showed that maternal weight changes usually occur after the first trimester [1]. Several studies investigated the association between trimester-specific gestational weight gain with offspring health outcomes and concluded that gestational weight gain during the 2^nd^ and 3^rd^ trimester was associated with outcomes of interest [2,3]. Therefore, we focused on the weight gain during the 2^nd^ and 3^rd^ trimesters of pregnancy in our study, and treated maternal BMI during early pregnancy as indicator of maternal pre-pregnancy weight status. We explained indicators in Materials and Methods part.

*Lines 185-188 “Maternal BMI during the first trimester (<14 weeks) was used as a proxy for pre-pregnancy BMI in the present study to classify GWG, given that maternal weight changes usually occur after the first trimester.”*

*Lines 191-195 “We calculated weekly gestational weight gain (kg/week) during the second and third trimesters. We selected GWG velocity as a predictor because it is independent of gestational age. According to IOM and NHC guidelines, inadequate, adequate and excessive GWG were defined as below, within, and above the recommended range of GWG velocity during the second and third trimesters.”*

#7. Lines 169-171 could authors present this comparison in table?

*Response*: Thank you for the comment. Our study focused on gestational weight gain velocity during 2^nd^ and 3^rd^ trimesters among normal weight women, so the context only involved two sets of ranges: 0.35-0.50kg/week by the Institute of Medicine (IOM) and 0.26-0.48kg/week by the National Health Commission (NHC) of the People’s Republic of China.

#8. Table 2. is this in gram per week of weight gain? we think is more communicative if we use per trimester and with kilogram unit data presentation.

*Response*: Thank you for the comment. This data presentation is in kilogram per week during 2^nd^ and 3^rd^ trimesters. According to IOM and NHC guidelines, they both combined 2^nd^ and 3^rd^ trimesters when establish recommended ranges. We mainly compared the association between GWG under guidelines established by different cohorts and birth outcomes. Therefore, we calculated average weekly gestational gains during 2^nd^ and 3^rd^ trimesters and then defined inadequate, adequate, and excessive GWG according to IOM and NHC guidelines. Additionally, gestational weight gain per trimester is also of great importance, and further studies can focus on trimester weight gain to established more precise guidelines.

#9.Line 320 we suggest to each sub-topic of the discussion should be written in each sub-tittle.

*Response*: Thank you for the comment. We have now added sub-titles in the discussion.

*Lines 362 “Guidelines for gestational weight gain”*

*Lines 380 “Gestational weight gain and birth outcomes”*

*Lines 442“Limitations”*

#10. Lines 344-345 why all approaches guideline have different result?

*Response*: Thank you for the comment. No consensus has been reached on defining optimal GWG among pregnant women. These three guidelines were established in different settings and populations by different approaches as described in Introduction session. The IOM recommendation was constructed based on the lowest prevalence of the commonly pregnant outcomes among US white women. The NHC recommendation utilized data from a cohort including more than 100,000 pregnant Chinese women considering composite maternal and neonatal outcomes. Moreover, the weight-gain-for-gestational age z-score standards according to the Fetal Growth Longitudinal Study of the INTERGROWTH-21^st^ Project was conducted in a multicenter including eight geographically diverse urban regions in Brazil, China, India, Italy, Kenya, Oman, the United Kingdom, and the United States and only considered healthy pregnant women with normal weight. We have described setting, population and approach of each standard in the Introduction part. As shown in Table 2, there are much difference of GWG categories between IOM and NHC guidelines. GWG outside recommendation range published by NHC was associated with adverse birth outcomes even among women with normal BMI during early pregnancy in this cohort of Chinese women and infants. Null associations were observed for IOM and INTERGROWTH-21st Project guideline. Different approaches showed different results, and that is why we argued that GWG guideline should be established targeting at pregnant women in different settings/regions. We have discussed this topic in the Discussion part.

*Lines 335-341 “Likewise, population and regional differences contribute to various women’s body sizes and weight gain profiles [12]. In addition, regional socioeconomic, medical, and cultural factors also play a role [28]. Therefore, developing their own BMI classification criteria has been emphasized based on their own morbidity and mortality data [29], as for the recommended GWG range. Several studies explored their own optimal GWG ranges suitable and sensible for regional pregnant women by various approaches.”*

#11. Line 407 authors need to write the effort that had made to overcome the limitations during the analysis and data collection.

*Response*: Thank you for the comment. For each limitation, we have discussed the efforts had made to overcome. For the first limitation, we discussed potential bias and adjusted randomized regimens in the models.

*Lines 455-461 “In the present study, we mainly focused on the association between GWG and birth outcomes and we observed similar GWG velocity and z-scores generated from INTERGROWTH 21st Project by treatment arms (see S8 Table). Therefore, we treated randomized regimens as a covariable in the models. We recognized that GWG is on the causal pathway between nutrition supplementation and fetal growth and could mediate and/or modify the relation. Still, we failed to elucidate it thoroughly in our study.”*

For the second limitation, we adjusted gestational age during the first trimester in the models.

*Lines 464-466 “All these methods would be similarly affected by this misclassification. Besides, we also adjust the gestational age in the first trimester.”*

For the third limitation, we adjusted pre-pregnancy disease history in the models and applied E-values to assess unmeasured confounders.

*Lines 468-472 “Although we failed to take pregnant complications as confounders into models in the present study, pre-pregnant disease history including hypertension, diabetes, heart disease, and anemia was considered. Furthermore, we applied E-values to assess unmeasured confounders, which showed the robustness of our results.”*

References:

1. Fattah C, Farah N, Barry SC, O’Connor N, Stuart B, Turner MJ. Maternal weight and body composition in the first trimester of pregnancy. Acta Obstet Gynecol Scand. 2010;89:952–5.

2. Association of trimester-specific gestational weight gain with fetal growth, offspring obesity, and cardiometabolic traits in early childhood. Am J Obstet Gynecol. 2015;212:502.e1-14.

3. Huang A, Ji Z, Zhao W, Hu H, Yang Q, Chen D. Rate of gestational weight gain and preterm birth in relation to prepregnancy body mass indices and trimester: a follow-up study in China. Reprod Health. 2016;13:93.
