# Supplementary material for: Associations between gestational weight gain under different guidelines and adverse birth outcomes: A secondary analysis of a randomized controlled trial in rural western China
Source: PLOS Glob Public Health. 2024 Jan 8;4(1):e0002691. doi: 10.1371/journal.pgph.0002691 (PMC10773947; doi:10.1371/journal.pgph.0002691)
Supplement: Attachment — Submitted filename: 20231101 Response to Reviewers.docx [file pgph.0002691.s014.docx]

Manuscript Number: PGPH-D-23-00983

Title: Associations between gestational weight gain in different guidelines and adverse birth outcomes: a secondary analysis of a randomized controlled trial in rural western China

We heartily thank the editors and reviewers for their thoughtful comments and suggestions, which have helped to improve the quality of this manuscript.

Reviewer #2:

1. There is a lack of rationale explaining why different guidelines are associated with adverse birth outcomes. The rationale together with specific hypotheses, including why you are comparing two guidelines, should be clearly stated in the Introduction.

*Response*: Thank you for the comment. We have carefully revised the structure of Introduction. We have now provided more rationales for comparing different guidelines and specific hypothesis in our study area in the Introduction section.

*Lines 104-112 “However, whether the predetermined cutoffs in IOM guideline could minimize adverse maternal and neonatal outcomes among population residing in LMICs remains no consensus. A meta-analysis included studies from the USA and western Europe suggested that GWG above IOM guideline was associated with macrosomia (OR 1.87; 95% CI 1.70, 2.06) [10]. While, meta-analysis conducted among sub-Saharan Africa researches found no significantly association between excessive GWG and macrosomia after applying IOM guidelines [11]. The establishment of IOM was based on USA white women and mainly examined in developed settings, where the incidence of overweight and obesity is much higher than those women residing in LMICs [10].”*

*Lines 133-143 “Studies mentioned above were conducted in relatively developed cities in China and did not include the population in rural western areas. Health conditions of women in reproductive age varied across different socioeconomic groups and geographies in China. For example, the prevalence of anemia in women of reproductive age was 45.7% in rural western areas of China in 2005, while corresponding average prevalence was 19.9% in 2002 in China according to China Health and Nutrition Survey [18]. In addition, none investigation has examined the appliance of weight-gain-for-gestational age z-score constructed by the INTERGROWTH-21st Project among Chinese women. Therefore, whether the existing three GWG guidelines above could applied to the population in rural western areas in China and their associations with adverse birth outcomes remain unknown.”*

*Lines 152-156 “We hypothesized that the GWG recommend ranges released by NHC would be more suitable for Chinese women in rural western China. In addition, these results would help to expand the generalization of appropriate GWG guidelines for optimizing birth outcomes in other rural areas in LMICs.”*

2. How covariates were chosen should be described in more detail in the methods section. However, the operationalization of these variables should be provided in the text.

*Response*: Thank you for the comment. We considered the common confounders based on the literatures mainly including baseline socioeconomic characteristics, maternal nutrition status, and offspring sex. We adjusted all covariables in each model. We have now provided details on how the covariates were chosen and entered into the statistical models in the Methods section.

*Lines 219-221 “Common confounders were selected based on the literatures including parental sociodemographic characteristics and maternal nutrition status.”*

*Line 258“Confounder mentioned above were adjusted in each regression model.”*

3. The analysis was based on data from a longitudinal cohort study. What is the attrition rate? Attrition analysis should be conducted and discussed.

*Response*: Thank you for the comment. The present study is a secondary analysis of origin parent trial. To examine our study hypothesis, final analyses were limited to women of normal weight during early pregnancy and with singleton live birth. 4604 singleton births were obtained from the parental trial, while among them 2252 after 14 weeks of gestation age were not eligible for the analysis. Of the remaining 2352 participants, 688 women had missing values on weight data during the second or third trimester and 98 infants had missing values on birth weight or gestational age at birth. Among the remaining 1566 participants, 327 women with underweight or overweight/obesity were excluded from main analysis for the different cut-off points of normal weight using BMI by IOM and NHC guidelines. Finally, 1239 mother-infant pairs were included in the main analysis (flowchart as shown in Figure 1). The attrition rate is 73% [(4604-1239)/4604]. We compared the baseline characteristics between mother-infant pairs included in the main analysis and those were not eligible or excluded (as shown in Table 1 and 2). By comparing the sociodemographic characteristics of women enrolled during early pregnancy and those enrolled between 14 and 28 gestational weeks, we observed that women who were more educated or were non-farmer were more likely to be enrolled before 14 gestational weeks (Table 1). While, more than 80% women in each group were secondary education level and farmer. Moreover, we compared baseline characteristics between women included in the main analysis and those where not, we observed that women who were farmer, were more educated, had higher MUAC, or had higher household wealth were more likely to be included in the present analysis (Table 2). Thus, the proportion of inadequate GWG might be underestimate in the present study. To assess the potential selection bias, we conducted inverse probability weighting (IPW) analysis and the results showed similar association between suboptimal GWG classified by NHC and adverse outcome, except for macrosomia (Table 3). The percentage of macrosomia is as low as 1.5%, as well as the limited sample size, which might result in low power to assess the associations. Further, we discussed this limitation in the Discussion section. *Lines 487-496* “*Secondly, we limited analysis among those women enrolled less than 14 gestation weeks and without missing values of weight during the second or third trimesters which might lead to selection bias. We compared baseline characteristics of mother-infant pairs which enrolled in early pregnancy and those between more than 14 weeks’ gestation (see S Table 8). Women who were farmer, were more educated, had higher MUAC, or had higher household wealth were more likely to be included in the present analysis. Thus, the proportion of inadequate GWG might be underestimate in the present study. In addition, we applied IPW to consider the potential of selection bias, and the results showed consistently association of inadequate GWG with post-term birth and excessive GWG with LGA baby.”*

*Lines 244-251 “4,604 singleton births were obtained from the parental trial, while among them 2,252 after 14 weeks of gestation age were not available for the analysis. Of the remaining 2,352 participants, 688 women had missing values on weight data during the second or third trimester and 98 infants had missing values on birth weight or gestational age at birth. Among the remaining 1,566 participants, 327 women with underweight or overweight/obesity were excluded from main analysis for the different cut-off points of normal weight using BMI by IOM and NHC guidelines. Finally, 1,239 mother-infant pairs were included in the main analysis (as shown in S Figure 1).”*

*Lines 272-276 “For supplementary analyses, we compared the baseline characteristics between women included in the main analysis and those were not. Further, we conducted inverse probability weighting (IPW) to address the potential of selection bias due to the missing value of maternal weight and birth outcomes. IPW was constructed using covariables mentioned above.”*

*Lines 366-371“By comparing the baseline characteristics between women included in the main analysis and those where not, we observed that women who were farmer, were more educated, had higher MUAC, or had higher household wealth were more likely to be included in the present analysis (see S Table 8). After applying IPW, the results revealed similar associations of inappropriate GWG defined by NHC and adverse birth outcomes (similar to Table 3 and S Table 6).”*

Table 1. Comparison of baseline characteristics of mother-infant pairs between pregnant women enrolled before 14 gestational weeks and those enrolled between 14 and 28 gestational weeks.

| Factors | Enrolled in early pregnancy | Enrolled after early pregnancy | *P* |
| --- | --- | --- | --- |
| N | 2352 | 2252 |  |
| *Pregnant women characteristics* |  |  |  |
| Maternal age (years) /Mean (SD) | 24.6 (4.4) | 24.6 (4.2) | 0.65 |
| Maternal education /N (%) |  |  | 0.01 |
| < 3 years | 132 (5.6) | 129 (5.8) |  |
| Primary | 616 (26.2) | 591 (26.4) |  |
| Secondary | 1223 (52.1) | 1235 (55.2) |  |
| High school and above | 377 (16.1) | 283 (12.6) |  |
| Maternal occupation /N (%) |  |  | 0.01 |
| Farmer | 1930 (82.5) | 1908 (85.5) |  |
| Others | 410 (17.5) | 325 (14.6) |  |
| Parity at enrollment /N (%) |  |  | 0.40 |
| 0 | 1520 (64.6) | 1478 (65.6) |  |
| 1 | 746 (31.7) | 680 (30.2) |  |
| ≥2 | 86 (3.7) | 94 (4.2) |  |
| Maternal MUAC (cm) /N (%) |  |  | 0.79 |
| <21.5 | 432 (18.5) | 403 (18.2) |  |
| ≥21.5 | 1904 (81.5) | 1812 (81.8) |  |
| Randomized regimens /N (%) |  |  | 0.66 |
| Folic acid | 836 (35.5) | 800 (35.5) |  |
| Folic acid plus iron | 778 (33.1) | 721 (32.0) |  |
| Multiple micronutrient | 738 (31.4) | 731 (32.5) |  |
| Pre-pregnancy medical history /N (%) |  |  | 0.40 |
| Yes | 478 (20.6) | 433 (19.6) |  |
| No | 1842 (79.4) | 1776 (80.4) |  |
| Household wealth at enrollment^a^ /N (%) |  |  | 0.05 |
| Low | 733 (31.2) | 744 (33.0) |  |
| Medium | 804 (34.2) | 705 (35.8) |  |
| High | 815 (34.6) | 703 (31.2) |  |
| Offspring sex /N (%) |  |  | 0.21 |
| Male | 1283 (54.5) | 1270 (56.4) |  |
| Female | 1069 (45.5) | 982 (43.6) |  |
| Infant birth weight (gram) /Mean (SD) | 3183 (429) | 3192 (423) | 0.53 |
| Infant gestational age at birth/Mean (SD) | 39.8 (1.7) | 39.8 (1.9) | 0.61 |

Abbreviations: SD: standard deviations; MUAC, mid-upper arm circumference.

^a^Household wealth at enrollment was derived from principal component analysis for household assets and dwelling characteristics, and was further categorized by its terciles.

Table 2. Comparison of baseline characteristics between mother-infant pairs included into final analysis and those were not.

| Factors | Included in final analysis | Not included in the final analysis | *P* |
| --- | --- | --- | --- |
| N | 1239 | 3365 |  |
| Maternal age (years) /Mean (SD) | 24.5 (4.5) | 24.7 (4.3) | 0.12 |
| Maternal education /N (%) |  |  | 0.04 |
| < 3 years | 63 (5.1) | 201 (6.0) |  |
| Primary | 301 (24.4) | 908 (27.1) |  |
| Secondary | 670 (54.2) | 1786 (53.3) |  |
| High school and above | 202 (16.3) | 457 (13.6) |  |
| Maternal occupation /N (%) |  |  | <0.001 |
| Farmer | 1000 (81.2) | 2568 (76.5) |  |
| Others | 231 (18.8) | 787 (23.5) |  |
| Parity at enrollment /N (%) |  |  | 0.63 |
| 0 | 817 (65.9) | 2183 (64.8) |  |
| 1 | 378 (30.5) | 1049 (31.2) |  |
| ≥2 | 44 (3.5) | 135 (4.0) |  |
| Maternal MUAC (cm) /N (%) |  |  | <0.001 |
| <21.5 | 171 (13.9) | 664 (20.0) |  |
| ≥21.5 | 1062 (86.1) | 2656 (80.0) |  |
| Randomized regimens /N (%) |  |  | 0.06 |
| Folic acid | 456 (36.8) | 1182 (35.1) |  |
| Folic acid plus iron | 421 (34.0) | 1078 (32.0) |  |
| Multiple micronutrient | 362 (29.2) | 1107 (32.9) |  |
| Pre-pregnancy medical history /N (%) |  |  | 0.95 |
| Yes | 242 (19.7) | 651 (19.6) |  |
| No | 985 (80.3) | 2665 (80.4) |  |
| Household wealth at enrollment^a^ /N (%) |  |  |  |
| Low | 376 (30.4) | 1106 (32.8) | 0.03 |
| Medium | 418 (33.7) | 1191 (35.4) |  |
| High | 445 (35.9) | 1070 (31.8) |  |
| Offspring sex /N (%) |  |  | 0.73 |
| Male | 693 (55.9) | 1864 (55.4) |  |
| Female | 546 (44.1) | 1503 (44.6) |  |
| Infant birth weight (gram) /Mean (SD) | 3189 (411) | 3184 (434) | 0.73 |
| Infant gestational age at birth/Mean (SD) | 39.8 (1.5) | 39.8 (1.8) | 0.71 |

Abbreviations: SD: standard deviations; MUAC, mid-upper arm circumference.

^a^Household wealth at enrollment was derived from principal component analysis for household assets and dwelling characteristics, and was further categorized by its terciles.

Table 3. Sensitivity analysis using inverse probability weighting to assess the potential of selection bias of the association between different GWG classifications and adverse birth outcomes among women with underweight, normal, overweight or obesity (n=1566).

|  | IOM category^a^ | | NHC category^a^ | |
| --- | --- | --- | --- | --- |
|  | Inadequate | Excessive | Inadequate | Excessive |
| Preterm birth | 1.16 (0.55, 2.43) | 1.16 (0.452.98) | 1.36 (0.69, 2.70) | 1.20 (0.51, 2.83) |
| Post-term birth | 2.99 (1.30, 6.87) | 1.80 (0.66, 4.92) | 2.37 (1.31, 4.30) | 1.30 (0.60, 2.85) |
| LBW | 1.20 (0.51, 2.81) | 0.94 (0.33, 2.70) | 1.05 (0.54, 2.04) | 0.74 (0.31, 1.80) |
| Macrosomia | 2.65 (0.56, 12.53) | 3.13 (0.57, 17.13) | 1.09 (0.30, 3.97) | 1.86 (0.50, 6.93) |
| SGA | 1.27 (0.82, 1.97) | 1.16 (0.68, 2.01) | 1.20 (0.83, 1.73) | 0.99 (0.62, 1.59) |
| LGA | 1.18 (0.5, 2.55) | 2.22 (0.97, 5.08) | 1.05 (0.54, 2.03) | 2.18 (1.12, 4.25) |

Abbreviations: GWG, gestational weight gain; IOM, Institute of Medicine; NHC, National Health Commission; LBW, low birth weight; SGA, small-for-gestational-age; LGA, large-for-gestational-age; NA, not applicable.

^a^Data are presented with adjusted odd ratios and 95% confidence intervals by performing generalized linear models. The adjustments included parental education, occupation and age, maternal parity, the gestational week during early trimester when the maternal weight was measured, mid-upper arm circumference and randomized regimens, household wealth at enrollment, and infant sex.

4. Also clearly mention about co-morbid condition such as cardiac disease, hypertension, diabetes, tuberculosis, kidney disease, chronic liver disease, hyperthyroidism and or anemia in line number 200-202.

*Response*: Thank you for the suggestion. Pre-pregnancy medical history was obtained by face-to-face questionnaire at enrollment. For example, women were asked “Have you ever been diagnosed as cardiac disease? ”. The similar questions were used to collect information on tuberculosis, kidney disease, chronic liver disease, hypertension, diabetes, anemia or hyperthyroidism. The most prevalent disease was anemia, with a prevalence of 19.13%, while other diseases had a very low prevalence, ranging between 0.08% and 0.40% (see Table 4 below). In our sample, only nine women suffered from two diseases (three women with cardiac disease and anemia, three with kidney disease and anemia, two women with chronic liver disease and anemia, one woman with hypertension and anemia). One woman suffered from chronic liver disease, hypertension, and hyperthyroidism (as Table 4 shown below). Therefore, due to the low frequency of other diseases except for anemia, women having any one of these diseases was considered to have a preconception medical history for simplicity and convergence of statistical models, with a prevalence of 20% (242/1239). We have now added more details on the co-morbid conditions in Methods section.

*Lines 231-241 “The most prevalent disease during pregnancy was anemia in our sample, with a prevalence of 19.13% among women with normal weight, while other disease had a very low prevalence, ranging between 0.08% and 0.40%. In our sample, only nine women suffered from two diseases (three women with cardiac disease and anemia, three with kidney disease and anemia, two women with chronic liver disease and anemia, one woman with hypertension and anemia). Only one woman suffered from chronic liver disease, hypertension, and hyperthyroidism. Therefore, due to the low frequency of other disease except for anemia, women have any one of these cardiac disease, kidney disease, chronic liver disease, hypertension, anemia or hyperthyroidism, was considered to have a preconception medical history for simplicity and convergence of statistical models.”*

Table 4. The description of women with pre-pregnancy medical history.

|  | Case (n) | Percentage (%) |
| --- | --- | --- |
| cardiac disease | 4 | 0.32 |
| kidney disease | 3 | 0.24 |
| chronic liver disease | 5 | 0.40 |
| hypertension | 2 | 0.16 |
| anemia | 237 | 19.13 |
| hyperthyroidism | 1 | 0.08 |

Table 5. The description of women with comorbidity.

| Comorbidity combination | | | | | | case | Percentage (%) |
| --- | --- | --- | --- | --- | --- | --- | --- |
| cardiac disease | kidney disease | chronic liver disease | hypertension | anemia | hyperthyroidism |  |  |
| √ |  |  |  | √ |  | 3 | 0.24 |
|  | √ |  |  | √ |  | 3 | 0.24 |
|  |  | √ |  | √ |  | 2 | 0.16 |
|  |  |  | √ | √ |  | 1 | 0.08 |
|  |  | √ | √ |  | √ | 1 | 0.08 |

5. In discussion, line number 320-325 it’s a repetition of result and second para, line number 329-325 its not link with whole discussion. Need more justification why we need this study.

*Response*: Thank you for the comment. You might read the first version of our manuscript instead of the reversion. We apologize for any confusion or repetition in our discussion. We have now revised the structure of the Discussion section. We removed the repetition sentences of results and concluded our findings in the first paragraph of discussion and the contents which were not linked with the whole discussion. In addition, the justification for the importance of this study were included in the Introduction.

*Lines 377-386 “This study found that half of the pregnant women with normal weight in rural western China gained gestational weight below the recommendation for either of the IOM or NHC guideline. The kappa value for the classification agreement between IOM and NHC guidelines was 0.68. After applying NHC guideline, inadequate GWG classified was positively associated with post-term birth, and excessive GWG was associated with higher risk of macrosomia and LGA compared with adequate GWG. When applied weight-gain-for-gestational-age z-score derived from the INTERGROWTH-21^st^ Project, z-score was positively associated with birthweight while no significant association was observed between predefined subgroups of z-score and adverse birth outcomes.”*

*Lines 389-401 “IOM guideline has been the most widely adopted for researches and clinical practices in many countries. As the increasing evidence examined the utility of IOM guideline across continents and ethnicities, especially in LIMCs settings [10, 11], several studies aimed to explore optimal GWG ranges suitable for their regional population. In China, Zhang and colleagues recruited 3,731 women with singleton pregnancy from nine cities from 2013 to 2014 to establish optimal GWG ranges, which was defined as the range that did not exceed a 1% increase from the lowest predicted probability in each Chinese-specific pre-pregnancy BMI group [24]. Sun and colleagues enrolled 7976 pregnant women from 24 hospitals in 15 provinces of China from 2017 to 2018 and then derived appropriate total GWG ranges for each Chinese-specific BMI group [14]. However, their samples did not include the population in rural western areas in China, and cut-offs proposed above have not been formally issued to guide pregnant women weight management clinically.”*

*Lines 133-156 “Studies mentioned above were conducted in relatively developed cities in China and did not include the population in rural western areas. Health conditions of women in reproductive age varied across different socioeconomic groups and geographies in China. For example, the prevalence of anemia in women of reproductive age was 45.7% in rural western areas of China in 2005, while corresponding average prevalence was 19.9% in 2002 in China according to China Health and Nutrition Survey [18]. In addition, none investigation has examined the appliance of weight-gain-for-gestational age z-score constructed by the INTERGROWTH-21^st^ Project among Chinese women. Therefore, whether the existing three GWG guidelines above could applied to the population in rural western areas in China and their associations with adverse birth outcomes remain unknown.*

*In the present study, we used data from a cluster-randomized, double-blind trial in rural western China, where populations suffered from relatively disadvantaged socioeconomic status, with limited health workforce, health infrastructure, and maternal care [19]. We aimed to examine the association between GWG classifications and adverse birth outcomes among rural western Chinese mother-infant pairs under the NHC and IOM guidelines, respectively. In addition, we aimed to explore the association between the weight-gain-for-gestational-age z-scores constructed by the INTERGROWTH-21^st^ Project and birth outcomes. We hypothesized that the GWG recommend ranges released by NHC would be more suitable for Chinese women in rural western China. In addition, these results would help to expand the generalization of appropriate GWG guidelines for optimizing birth outcomes in other rural areas in LMICs.”*

Figure 1. Flowchart.
